# Substituent Effects on the Stability of Thallium and Phosphorus Triple Bonds: A Density Functional Study

**DOI:** 10.3390/molecules22071111

**Published:** 2017-07-05

**Authors:** Jia-Syun Lu, Ming-Chung Yang, Ming-Der Su

**Affiliations:** 1 Department of Applied Chemistry, National Chiayi University, Chiayi 60004, Taiwan; s1022818@mail.ncyu.edu.tw (J.-S.L.); mingchungmc@gmail.com (M.-C.Y.); 2 Department of Medicinal and Applied Chemistry, Kaohsiung Medical University, Kaohsiung 80708, Taiwan

**Keywords:** triply bonded molecules, triple bond, acetylene, substituent effects

## Abstract

Three computational methods (M06-2X/Def2-TZVP, B3PW91/Def2-TZVP and B3LYP/LANL2DZ+dp) were used to study the effect of substitution on the potential energy surfaces of RTl≡PR (R = F, OH, H, CH_3_, SiH_3_, SiMe(Si*t*Bu_3_)_2_, Si*i*PrDis_2_, Tbt (=C_6_H_2_-2,4,6-(CH(SiMe_3_)_2_)_3_), and Ar* (=C_6_H_3_-2,6-(C_6_H_2_-2, 4,6-*i*-Pr_3_)_2_)). The theoretical results show that these triply bonded RTl≡PR compounds have a preference for a bent geometry (i.e., ∠R⎼Tl⎼P ≈ 180° and ∠Tl⎼P⎼R ≈ 120°). Two valence bond models are used to interpret the bonding character of the Tl≡P triple bond. One is model [I], which is best described as Tl
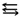
P. This interprets the bonding conditions for RTl≡PR molecules that feature small ligands. The other is model [II], which is best represented as Tl
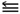
P. This explains the bonding character of RTl≡PR molecules that feature large substituents. Irrespective of the types of substituents used for the RTl≡PR species, the theoretical investigations (based on the natural bond orbital, the natural resonance theory, and the charge decomposition analysis) demonstrate that their Tl≡P triple bonds are very weak. However, the theoretical results predict that only bulkier substituents greatly stabilize the triply bonded RTl≡PR species, from the kinetic viewpoint.

## 1. Introduction

The preparation and characterization of triply bonded heavier main group element (E_14_ = Si, Ge, Sn, and Pb) molecules (i.e., RE_14_≡E_14_R) is a popular field of study in inorganic chemistry [1,2,3,4,5,6,7,8,9,10,11,12,13,14,15,16,17,18,19,20,21,22,23,24,25,26,27,28,29,30,31,32,33,34,35,36,37,38,39,40,41]. From the valence electron viewpoint, the triply bonded RE_13_≡E_15_R compound is isoelectronic to the RE_14_≡E_14_R species. However, the former has been the subject of much less study than the latter, in the field of synthetic chemistry. Therefore, the level of understanding of the chemistry of RE_13_≡E_15_R is lower than that for group 14 less-coordinate alkyne analogues.

In the group 15 family, phosphorus is more similar to its diagonal relative, carbon, than to nitrogen [42]. Thallium is also known to be monovalent and has an ionic radius that is similar to that of potassium, so it is often presumed to be a pseudo alkali metal [43]. The isolation and characterization of the singly bonded organothallium phosphorus molecule, (Me_3_SiCH_2_)_3_Tl⎼P(SiMe_3_)_3_, was experimentally reported about twenty years ago [44]. Two other novel compounds that contain the thallium⎼phosphorus single bond have also been identified [45,46]. If both thallium and phosphorus elements could be stabilized using a single bond to connect them, it might be possible to extend this field to the study of other triply bonded RTl≡PR inorganic molecules. This work reports the first theoretical study of the possible synthesis of the RTl≡PR molecule, which may be isolable as a long-lived compound. The study determines potential inorganic complexes that can stabilize the thallium≡phosphorus triple bond, to demonstrate the theoretical possibility that these unusual acetylene inorganic analogues can be synthesized.

## 2. Methodology

Using the Gaussian 09 program package [47], all geometries are fully optimized at the M06-2X [48], B3LYP [49,50], and B3PW91 [51,52] levels of theory, in conjunction with the Def2-TZVP [53] and LANL2DZ+dp [54,55,56,57,58] basis sets. These DFT calculations are signified as M06-2X/Def2-TZVP, B3PW91/Def2-TZVP and B3LYP/LANL2DZ+dp, respectively. In order to confirm that the reactants and products have no imaginary frequencies and that the transition states possess only one imaginary frequency, frequency calculations were performed for all structures. Thermodynamic corrections to 298 K, heat capacity corrections and entropy corrections (ΔS) are applied to the three levels of DFT. The relative free energy (ΔG) at 298 K is also computed at the same levels of theory.

Next, (Si*i*PrDis_2_)Tl≡P(Si*i*PrDis_2_), (Tbt)Tl≡P(Tbt), and (Ar*)Tl≡P(Ar*) are the model reactants for this study. It is known that the B3LYP functional fails to describe non-valent interactions, such as the London dispersion correctly. As a result, for large ligands, calculations were performed using dispersion-corrected M06-2X method [48]. Because of the limitations of the available memory size and CPU time, frequencies are not computed at the dispersion-corrected M06-2X/Def2-TZVP level of theory for the triply bonded R´Tl≡PR´ systems that have bulky ligands (R´), so the zero-point energies and the Gibbs free energies that are derived using the dispersion-corrected M06-2X/Def2-TZVP cannot be used for these systems.

## 3. General Considerations

Two interaction models that describe the chemical bonding of the triply bonded RTl≡PR, which serve as a basis for discussion, are given in this section. For convenience, the RTl≡PR molecule is divided into two fragments: Tl⎼R and P⎼R. On the basis of theoretical results (see below), three computational methods (M06-2X/Def2-TZVP, B3PW91/Def2-TZVP and B3LYP/LANL2DZ+dp) all indicate that the Tl⎼R and P⎼R fragments are respectively calculated to be in the singlet ground state and the triplet ground state.

**Figure 1:**
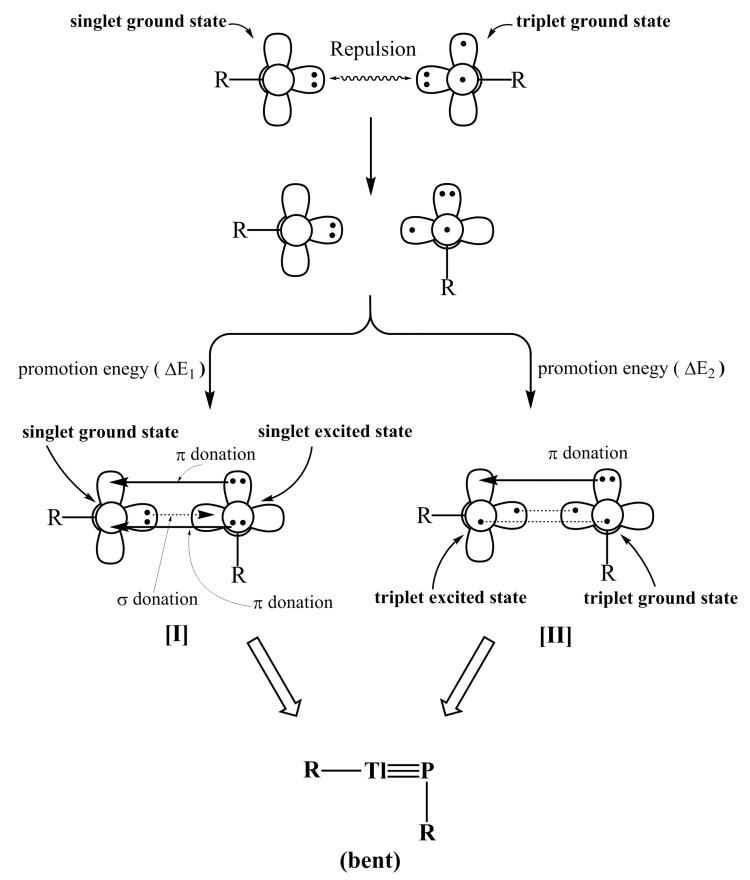
The interaction models, [I] and [II], for the triply bonded RTl≡PR molecule.

In model [I], electron promotion energy (ΔE_1_) forces the P⎼R moiety from the triplet ground state to the singlet excited state, so the electronic structure of RTl≡PR can be described in terms of the dimerization of singlet Tl⎼R and singlet P⎼R fragments, as shown in Figure 1. From the chemical bonding viewpoint, model [I] shows that the Tl≡P triple bond consists of one σ⎼donation of Tl→P and two π⎼donations of Tl←P. In model [II], the electron advancement energy (ΔE_2_) promotes the Tl⎼R unit from the singlet ground state to the triplet excited state. Accordingly, the bonding structure of RTl≡PR can also be represented as the dimerization of triplet Tl⎼R and triplet P⎼R fragments, as shown in Figure 1. From the bonding structure viewpoint, model [II] shows that the Tl≡P triple bond is composed of one Tl←P π⎼bond, one regular σ⎼bond and one π⎼bond.

It is schematically shown in Figure 1 that the formation of the triply bonded RTl≡PR molecule can be regarded as either [Tl⎼R]^1^ + [P⎼R]^1^ → [RTl≡PR]^1^ (model [I]) or [Tl⎼R]^3^ + [P⎼R]^3^ → [RTl≡PR]^1^ (model [II]). It is worthy of note that since the lone pair of phosphorus has significant amount of s character, this could reduce the bonding overlaps between Tl and P elements (see the black lines in model [I] and model [II] in Figure 1). As a consequence, the Tl≡P triple bond should be very weak, which is in contrast to the traditional triple bond of acetylene. This prediction is confirmed in the following section. Both models are used in this study clearly show that the Tl≡P triple bond is mostly attributed to electron donation from the lone pair of P to the empty p-orbital of Tl.

This bonding analysis is used to interpret the bonding properties of the triply bonded RTl≡PR molecule in the next section.

## 4. Results and Discussion

### 4.1. Small Ligands on Substituted RTl≡PR

The effect of small substituents on the stability of the triply bonded RTl≡PR species is discussed from the kinetic and the thermodynamic viewpoints. Five small substituents (R = H, F, OH, CH_3_ and SiH_3_) are used for the RTl≡PR model molecule. The important geometrical parameters for the RTl≡PR compounds are calculated at the three computational methods (M06-2X/Def2-TZVP, B3PW91/Def2-TZVP and B3LYP/LANL2DZ+dp) and the results are listed in Table 1. The Cartesian coordinates for the triply bonded minima are given in the Supplementary Information.

There are four noteworthy features of Table 1:

(1) The central Tl≡P triple bond distances (Å) for R = F, OH, H, CH_3_ and SiH_3_ are respectively estimated to be 2.313⎼2.422 Å, 2.336⎼2.443 Å and 2.331⎼2.480 Å, at the M06-2X/Def2-TZVP, B3PW91/Def2-TZVP and B3LYP/LANL2DZ+dp levels of theory. As mentioned in the Introduction, neither experimental nor theoretical results for the triply bonded RTl≡PR species are available to allow a definitive comparison. However, to the author’s best knowledge, there are only a few published reports concerning the singly bonded R_3_Tl⎼PR_3_ molecules and these report the Tl⎼P bond length to be 2.922 Å [44], 3.246–3.301 Å [45] and 3.032–3.168 Å [46]. These single bond distances are all longer than the sum of the covalent radii (i.e., 2.62 Å) [62] for the Tl and P elements.

(2) The three DFT calculations shown in Table 1 demonstrate that the R⎼Tl and R⎼P components have a singlet and triplet ground state, respectively. The three DFT computational results also show that the singlet-triplet energy differences (ΔE_ST_) for R⎼Tl and R⎼P fragments are estimated to be at least +67 and −15 kcal/mol, respectively. These energy values strongly suggest that model [I], which is shown in Figure 1, is superior to model [II] in describing the bonding characters of triply bonded RTl≡PR molecules that feature small substituents (R). Model [I] shows that the bonding structure of the triple bond in RTl≡PR can be represented as Tl
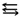
P. It must be noted that the fact that the lone pair of phosphorus has s character and the valence p orbital of phosphorus is much smaller than that of thallium means that both factors can vigorously affect the bonding overlaps between phosphorus and thallium atoms. Therefore, it is anticipated that the triple bond in these RTl≡PR species is very weak. This prediction is confirmed by the three DFT calculations shown in Table 1. All of the values for the Wiberg bond index (WBI) [59,60,61] are a little bit higher than 1.0, rather than 2.0. That is to say, regardless of whether small electropositive or small electronegative groups are attached, the RTl≡PR systems possess a quite weak Tl≡P triple bond.

(3) As already shown, model [I] describes the bonding characters in triply bonded RTl≡PR compounds that feature small substituents better than model [II]. This, in turn, strongly implies that an acute bond angle ∠Tl⎼P⎼R (close to 90°) and a linear bond angle ∠R⎼Tl⎼P (close to 180°) is favored in the triply bonded RTl≡PR molecule, which is verified by the three DFT calculations as shown in Table 1. The nearly perpendicular angle on the P center can also be attributed to the “orbital non-hybridization effect” [63,64,65,66] and the “inert *s*-pair effect” [63,64,65,66] as discussed previously.

(4) The binding energies (BE) that are required to cleave the central Tl≡P bond, which leads to one R⎼Tl and one R⎼P fragment in the singlet ground state and in the triplet ground state, respectively, are summarized in Table 1. The calculated BE values (kcal/mol) for the RTl≡PR molecules are in the range of 67–96, 67–96 and 68–93, at the M06-2X, B3PW91 and B3LYP levels of theory, respectively. This data confirms that the central thallium and phosphorus atoms in the substituted RTl≡PR compounds are strongly bonded.

**Figure 2:**
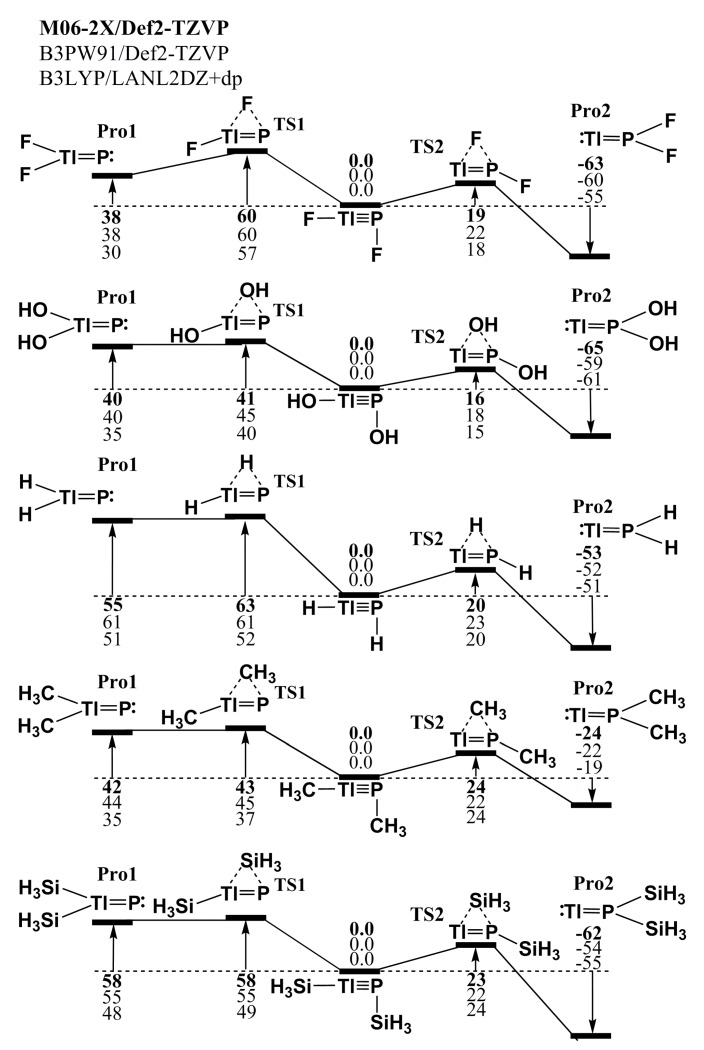
The Relative Gibbs free energy surfaces for RTl≡PR (R = F, OH, H, CH3 and SiH3). These energies are in kcal/mol and are calculated at the M06-2X/Def2-TZVP, B3PW91/Def2-TZVP, and B3LYP/LANL2DZ+dp levels of theory. For details see the text and Table 1.

Considering the stability of RTl≡PR, the theoretical results for the potential energy surfaces of the model molecule, RTlPR (R = F, OH, H, CH_3_ and SiH_3_), are described in Figure 2. This figure shows a number of stationary points exist, including local minima that correspond to RTl≡PR, R_2_Tl=P, Tl=PR_2_ and the transition states that connect them. The three DFT computational results show that all of the triply bonded RTl≡PR compounds that feature small substituents immediately transfer to the corresponding doubly bonded species via facile 1,2-migration reactions. In other words, the theoretical evidence shows that triply bonded RTl≡PR species that feature small ligands are both kinetically and thermodynamically unstable, regardless of whether they are electronegative or electropositive, so it is unlikely that they could be prepared or synthesized in a laboratory.

### 4.2. Large Ligands on Substituted R′Tl≡PR′

**Figure 3:**
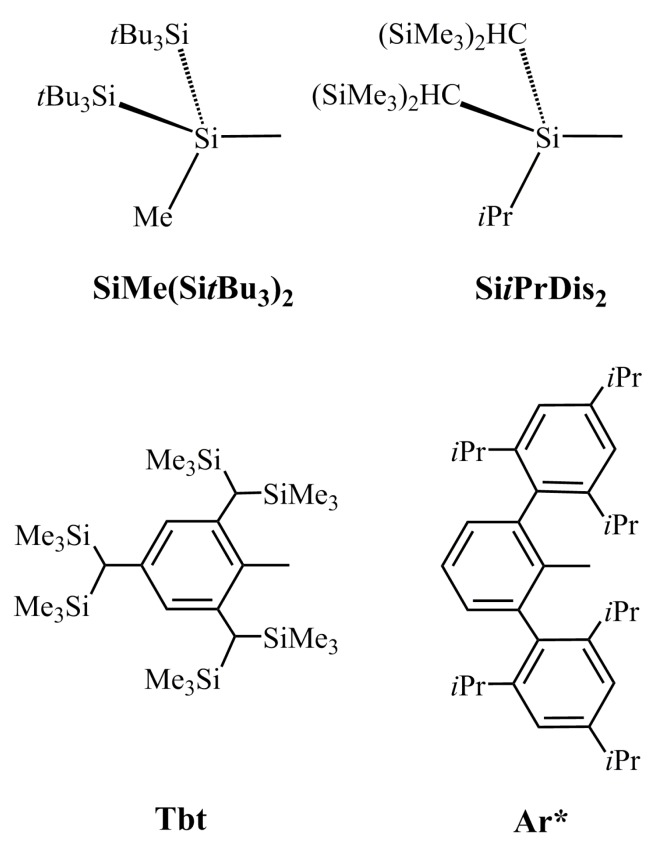
Four bulky groups. For details, see references [66,67].

As previously mentioned, in order to stabilize R′Tl≡PR′ from the kinetic viewpoint, three types of large substituents (R´) are used in this study. These are SiMe(Si*t*Bu_3_)_2_, Si*i*PrDis_2_, Tbt (=C_6_H_2_-2,4,6-(CH(SiMe_3_)_2_)_3_), and Ar* (=C_6_H_3_-2,6-(C_6_H_2_-2,4,6-*i*-Pr_3_)_2_) [67,68], as shown in Figure 3. The geometrical structures of R′Tl≡PR′ are optimized at the dispersion-corrected M06-2X/Def2-TZVP [53] level of theory. Their important calculated parameters are listed in Table 2.

Five important conclusions can be drawn from these theoretical results:

(i) The results presented in Table 2 predict that the Tl≡P triple bond lengths (Å) are about 2.386 Å, 2.384 Å, 2.385 Å, and 2.336 Å, for (SiMe(Si*t*Bu_3_)_2_)Tl≡P(SiMe(Si*t*Bu_3_)_2_), (Si*i*PrDis_2_)Tl≡P(Si*i*PrDis_2_), (Tbt)Tl≡P(Tbt), and (Ar*)Tl≡P(Ar*), respectively. These theoretically estimated values are shorter than the experimentally reported Tl⎼P single bond distance, as mentioned previously [44,45,46]. Similarly to the case for small substituents, the DFT optimized results show that all of the triply bonded R′Tl≡PR′ molecules that feature bulky ligands studied adopt a bent structure, as shown in Table 2.

(ii) If the R´Tl≡PR´ compound is cut in half, the Tl⎼R´ and P⎼R′ two fragments are obtained. The DFT results shown in Table 2 demonstrate that the ΔE_ST_ for the Tl⎼R′ unit is greater than 30 kcal/mol and the modulus of ΔE_ST_ for the P⎼R′ moiety is greater than 37 kcal/mol. That is to say, the promotion energy from the singlet ground state to the triplet excited for Tl⎼R′ is smaller than the energy that is required for promotion from that for Tl⎼R (Table 1). The bonding model that is shown in Figure 1 shows that model [II] can be used to interpret the bonding character in triply bonded R′Tl≡PR′ molecules that feature bulky ligands, R′. Namely, the bonding structure of the triple bond in R′Tl≡PR′ is best described as Tl
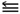
P. In this model, the electrons that are donated from the lone pair of phosphorus have s character, as shown in Figure 1. Moreover, the size of 2p orbital of P is also much smaller than the 6p orbital of Tl. These two factors combined produce a weak Tl≡P triple bond in the R′Tl≡PR′ species. Supporting theoretical evidence in Table 2 shows that the WBI for R′Tl≡PR′ is 2.21, 2.37, 2.13, and 2.20 for R = SiMe(Si*t*Bu_3_)_2_, Si*i*PrDis_2_, Tbt, and Ar*, respectively. These WBI values are much smaller than the value for acetylene (2.99).

(iii) In order to determine the effect of bulky substituents on the stability of triply bonded R′Tl≡PR′ compounds, the dispersion-corrected M06-2X/Def2-TZVP level of theory is used to determine the potential energy surfaces for the isomerization reaction. As shown in Table 2, the triply bonded R′Tl≡PR′ molecules have values that are at least 87 (ΔH_1_) and 71 (ΔH_2_) kcal/mol lower than that for the corresponding doubly bonded isomers. Therefore, the theoretical results show that a triply bonded R′Tl≡PR′ compound that features bulky substituents is more stable than its corresponding doubly bonded R′_2_Tl=P: and: Tl=PR′_2_ isomers, from the kinetic viewpoint.

(iv) In order to verify the conclusion from point (ii), “charge decomposition analysis” (CDA), reported by Dapprich and Frenking [69] is used in the present study. For instance, the computational results concerning (SiMe(Si*t*Bu_3_)_2_)Tl≡P(SiMe(Si*t*Bu_3_)_2_) based on the dispersion-corrected M06-2X/Def2-TZVP method are collected in Table 3. As seen in the X column, the biggest contribution from R′⎼Tl to R′⎼P is No.227 (HOMO⎼1) orbital. However, the largest contribution from R′⎼P to R′⎼Tl is No.228 (HOMO) orbital. As a result, the net electron transfer (−0.213) is from R′⎼P to R′⎼Tl, which is shown in the (X – Y) column. Namely, the R′⎼P unit donates more electrons to the R′⎼Tl unit. The theoretical evidence is in good agreement with the valence-electron bonding model (Figure 1; model [II]) as stated earlier. Consequently, the bonding nature of R′Tl≡PR′ can be considered as R′Tl
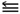
PR′.

**Figure 5:**
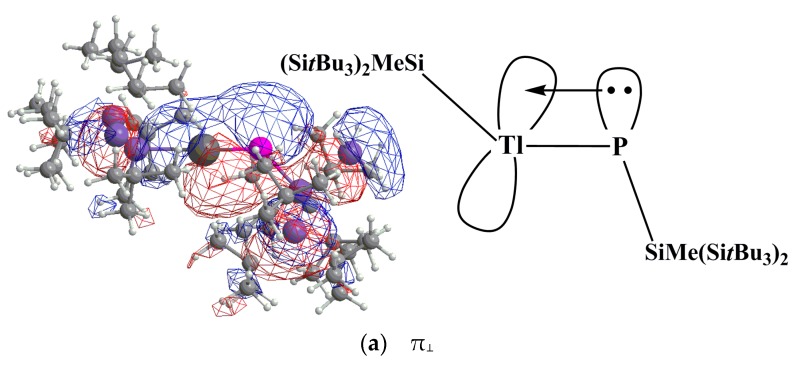

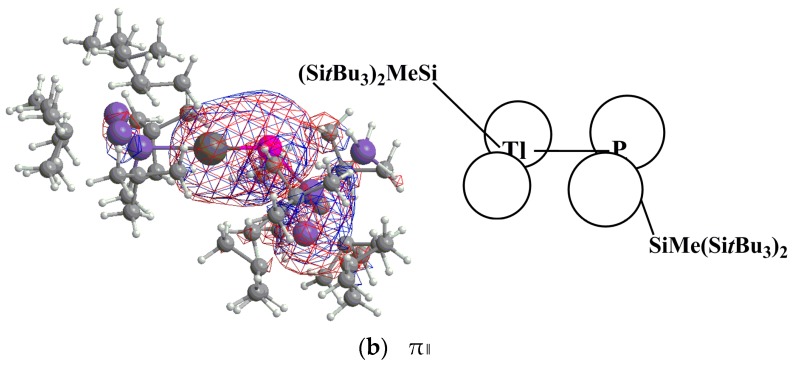
The natural Tl≡P π bonding orbitals ((**a**) and (**b**)) for (SiMe(Si*t*Bu_3_)_2_)Tl≡P(SiMe(Si*t*Bu_3_)_2_). For comparison, see also Figure 3.

(v) The NBO [59,60,61] and NRT [70,71,72] are also used to determine the bonding properties of the electronic structures of the R′Tl≡PR′ molecules, as shown in Table 4. This table clearly shows that the major bonding character between Tl and P comes from electron donation from 2p(P) to 6p(Tl), which is denoted as 6p(Tl) ← 2p(P). In the (SiMe(Si*t*Bu_3_)_2_)Tl≡P(SiMe(Si*t*Bu_3_)_2_) molecule, for instance, the dispersion-corrected M06-2X/Def2-TZVP calculations show that the Tl≡P π bonding occurs as follows: π_⊥_ (Tl≡P) = 0.3114(sp^4.77^)Tl + 0.9503(sp^1.42^)P. That is, a polarized π_⊥_ bond exists between Tl and P, which arises from the donation of the P lone pair to the empty Tl p orbital. As seen in Table 4, the Tl≡P π_⊥_ bonding orbitals comprise 9.7% natural Tl orbitals and 90% natural P orbitals (Figure 5). The similar theoretical results can also be found in the Tl≡P π_‖_ bonding orbitals as already represented in Table 4.

## 5. Conclusions

**Figure 6:**
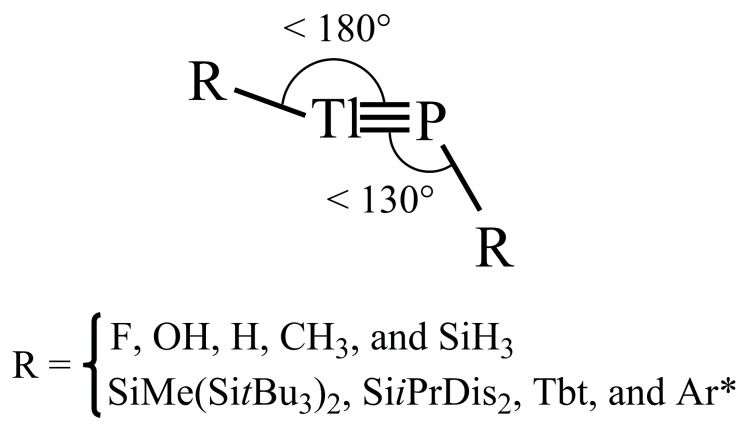
The predicted geometrical structure based on the present theoretical calculations.

In summary, the theoretical observations strongly support the idea that both electronic and steric effects determine the relative stability of molecules that contain a Tl≡P triple bond, as well as its corresponding doubly bonded isomers. The simple bonding models schematically illustrated in Figure 1 show that model [I], whose bonding character is symbolized by Tl
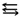
P, better interprets the triple bond in RTl≡PR species that feature small substituents. Model [II], whose bonding property is typified as Tl
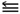
P, better describes the triple bond in R′Tl≡PR′ molecules that feature bulky ligands (Figure 6). However, regardless of whether the substituents in triply bonded RTl≡PR compound are large or small, their Tl≡P triple bonds are quite weak. Two effects can explain these phenomena. The different sizes of the p orbitals in the Tl and P elements mean that their overlapping populations are pretty small and the lone pair of the phosphorus atom has significant amount of s character, which results in poor overlaps between thallium and phosphorus. It is hoped that the results of experimental synthesis and structural characterization will confirm these predictions.

## Figures and Tables

**Figure 4:**
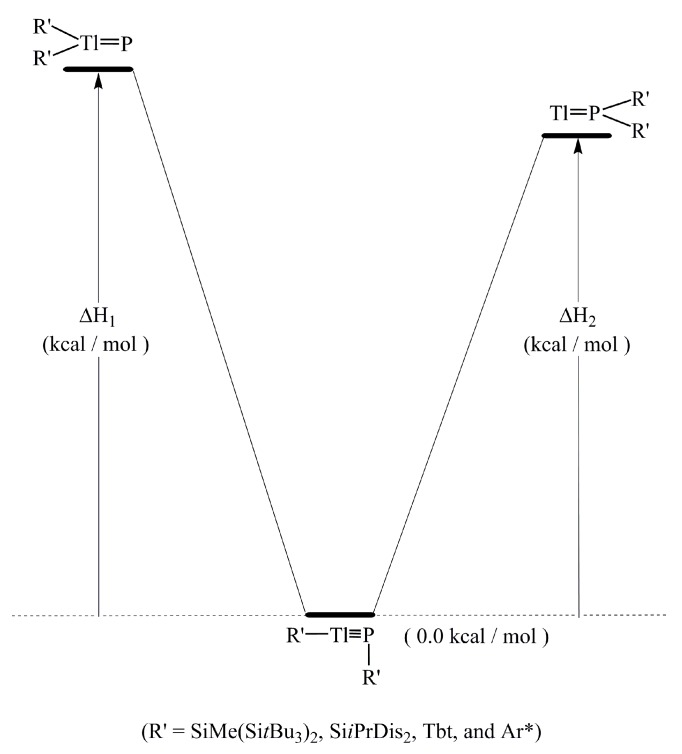
The potential energy surface for the 1,2-migration reaction of the R′Tl≡PR′ molecules with balky groups (R′).

**Table 1:** The important geometrical parameters, the natural charge densities (Q_Tl_ and QP), the binding energies (BE), the HOMO-LUMO energy gaps and the Wiberg Bond Index (WBI) for RTl≡PR using the M06-2X/Def2-TZVP, B3PW91/Def2-TZVP (in round brackets) and B3LYP/LANL2DZ+dp (in square brackets) levels of theory.

R	F	OH	H	CH_3_	SiH_3_
**Tl≡P (Å)**	2.422	2.437	2.320	2.339	2.313
	(2.425)	(2.443)	(2.327)	(2.349)	(2.336)
	[2.455]	[2.480]	[2.331]	[2.360]	[2.337]
**R-P-Tl (°)**	179.7	179.1	179.1	175.2	174.6
	(179.7)	(176.5)	(178.5)	(174.5)	(175.7)
	[178.5]	[177.9]	[178.2]	[171.3]	[179.1]
**P-Tl-R (°)**	94.63	98.92	86.51	100.4	94.76
	(96.59)	(101.5)	(86.82)	(102.2)	(92.71)
	[94.22]	[100.1]	[86.36]	[102.6]	[90.78]
**R-P-Tl-R (°)**	180.0	179.4	179.1	178.0	177.0
	(180.0)	(178.8)	(179.2)	(178.8)	(179.1)
	[180.0]	[179.2]	[179.8]	[179.9]	[179.4]
**Q_P_^(1)^**	0.16	0.076	−0.63	−0.37	−0.83
	(0.17)	(0.13)	(−0.60)	(−0.33)	(−0.72)
	[0.096]	[0.021]	[−0.62]	[−0.39]	[−0.76]
**Q_Tl_^(2)^**	1.19	1.14	1.12	1.07	0.82
	(1.11)	(1.03)	(0.87)	(0.99)	(0.75)
	[1.25]	[1.17]	[0.99]	[1.13]	[0.89]
**ΔE_ST_ for Tl⎼R (kcal/mol) ^(3)^**	102.1	83.57	84.85	66.82	75.96
	(103.7)	(80.69)	(85.69)	(67.38)	(77.63)
	[102.2]	[83.15]	[83.05]	[67.94]	[74.40]
**ΔE_ST_ for P⎼R (kcal/mol) ^(4)^**	−28.91	−17.53	−30.75	−26.43	−15.84
	(−33.35)	(−21.29)	(−35.49)	(−30.26)	(−18.68)
	[−31.76]	[−20.24]	[−33.16]	[−29.21]	[−14.46]
**HOMO—LUMO (kcal/mol)**	184.1	167.6	210.6	151.2	142.1
	(131.6)	(118.1)	(212.0)	(149.3)	(145.1)
	[182.5]	[169.1]	[215.4]	[146.5]	[148.5]
**BE (kcal/mol) ^(5)^**	95.58	83.57	84.85	66.82	75.96
	(95.74)	(82.10)	(85.69)	(67.38)	(77.63)
	[93.43]	[83.15]	[83.05]	[67.94]	[74.40]
**WBI ^(6)^**	1.159	1.162	1.456	1.382	1.404
	(1.194)	(1.197)	(1.491)	(1.415)	(1.417)
	[1.191]	[1.178]	[1.475]	[1.403]	[1.372]

^(1)^ The natural charge density on the central phosphorus atom; ^(2)^ The natural charge density on the central thallium atom; ^(3)^ ΔE_ST_ (kcal mol^−1^) = E(triplet state for R⎼Tl) – E(singlet state for R⎼Tl); ^(4)^ ΔE_ST_ (kcal mol^−1^) = E(triplet state for R⎼P) – E(singlet state for R⎼P); ^(5)^ BE (kcal mol^−1^) = E(singlet state for R⎼Tl) + E(triplet state for R⎼P) – E(singlet for RTl≡PR); ^(6)^ The Wiberg bond index (WBI) for the Tl≡P bond: see reference [59,60,61].

**Table 2:** The Bond Lengths (Å), Bond Angels (°), Singlet—Triplet Energy Splitting (Δ*E*ST), Natural Charge Densities (QTl and QP), Binding Energies (BE), the HOMO-LUMO Energy Gaps, the Wiberg bond index (WBI), and Some Reaction Enthalpies for R′Tl≡PR′ at the dispersion-corrected M06-2X/Def2-TZVP Level of Theory. See also Figure 4.

R′	SiMe(Si*t*Bu_3_)_2_	Si*i*PrDis_2_	Tbt	Ar*
**Tl≡P (Å)**	2.386	2.384	2.385	2.336
**∠R′–Tl–P (°)**	166.9	166.4	168.9	161.2
**∠Tl–P–R′ (°)**	122.3	113.7	116.2	115.6
**∠R′–Tl–P–R′ (°)**	171.4	179.5	173.9	174.4
**Q_Tl_^(1)^**	0.975	0.739	1.166	1.218
**Q_P_^(2)^**	−0.860	−0.826	−0.344	−0.257
**ΔE_ST_ for Tl—R′ (kcal/mol) ^(3)^**	35.91	35.52	31.27	30.24
**ΔE_ST_ for P—R′ (kcal/mol) ^(4)^**	−43.10	−37.47	−39.74	−40.52
**HOMO—LUMO (kcal/mol)**	71.27	27.21	58.05	39.34
**BE (kcal/mol) ^(5)^**	80.24	85.43	62.51	67.89
**ΔH_1_ (kcal/mol) ^(6)^**	91.34	90.49	89.22	87.11
**ΔH_2_ (kcal/mol) ^(6)^**	73.98	72.83	71.27	74.01
**WBI ^(7)^**	2.116	2.273	2.127	2.201

^(1)^ The natural charge density on the central thallium atom; ^(2)^ The natural charge density on the central phosphorus atom; ^(3)^ ΔE_ST_ (kcal mol^−1^) = E(triplet state for R′⎼Tl) – E(singlet state for R′⎼Tl); ^(4)^ ΔE_ST_ (kcal mol^−1^) = E(triplet state for R′⎼P) – E(singlet state for R′⎼P); ^(5)^ BE (kcal mol^−1^) = E(triplet state for R′⎼Tl) + E(singlet state for R′⎼P) – E(singlet for R′Tl≡PR′); ^(6)^ See Figure 4; ^(7)^ The Wiberg bond index (WBI) for the Tl≡P bond: see reference [59,60,61].

**Table 3:** The charge decomposition analysis (CDA) ^(a)^ for R′Tl≡PR′ (R′ = SiMe(Si*t*Bu_3_)_2_) system based on M06-2X orbitals, where the X term indicates the number of electrons donated from R′⎼Tl fragment to R′⎼P fragment, the Y term indicates the number of electrons back donated from R′⎼P fragment to R′⎼Tl fragment and the Q term indicates the number of electrons involved in repulsive polarization. Significant X and Y terms are bolded for easier comparison. ^(a),(b)^

	Orbital	Occupancy	X	Y	X – Y	Q
	218	2.000000	0.000757	0.000586	0.000171	−0.002462
	219	2.000000	0.001036	0.000522	0.000513	−0.004450
	220	2.000000	0.000932	0.000539	0.000394	−0.006342
	221	2.000000	0.000026	0.004350	−0.004325	−0.002504
	222	2.000000	0.001151	−0.000164	0.001315	−0.001354
	223	2.000000	0.000081	0.003145	−0.003064	−0.001960
	224	2.000000	0.000037	0.002403	−0.002366	−0.000054
	225	2.000000	0.001777	0.029263	−0.027486	−0.030329
	226	2.000000	0.000477	0.013735	−0.013259	−0.007124
	227	2.000000	0.008445	0.068258	−0.059813	−0.018272
HOMO	228	2.000000	−0.005339	0.003033	−0.008432	−0.004437
LUMO	229	0.000000	0.000000	0.000000	0.000000	0.000000
	230	0.000000	0.000000	0.000000	0.000000	0.000000
sum		456.000000	0.028853	0.241774	−0.212922	−0.107250

^(a)^ For clearness, only list the X, Y, and Q terms for HOMO (No.228) ⎼10 ~ LUMO+2. ^(b)^ Summation of contributions from all unoccupied and occupied orbitals.

**Table 4:** The natural bond orbital (NBO) and the natural resonance theory (NRT) analysis for R′Tl≡PR′ molecules that feature ligands (R′ = SiMe(Si*t*Bu_3_)_2_, Si*i*PrDis_2_, Tbt, and Ar*) at the dispersion-corrected M06-2X/Def2-TZVP level of theory ^(1,2)^.

R′Tl≡PR′	WBI	NBO Analysis			NRT Analysis	
		Occupancy	Hybridization	Polarization	Total/Covalent/Ionic	Resonance Weight
R′ = SiMe(Si*t*Bu_3_)_2_	2.11	σ = 2.21	σ : 0.5116 Tl (sp^1.27^) + 0.8592 P (sp^2.07^)	26.18% (Tl)	2.22/1.55/0.67	Tl⎼P: 23.17% Tl=P: 66.87% Tl≡P: 9.94%
				73.82% (P)		
		π_⊥_ = 1.84	π_⊥_: 0.3114 Tl (sp^4.77^) + 0.9503 P (sp^1.42^)	9.70% (Tl)		
				90.30% (P)		
		π_‖_ = 1.92	π_‖_: 0.6833 Tl (sp^99.87^) + 0.7556 P (sp^99.99^)	5.69% (Tl)		
				94.31% (P)		
R′ = Si*i*PrDis_2_	2.37	σ = 1.83	σ : 0.6422 Tl (sp^0.86^) + 0.7665 P (sp^20.18^)	41.24% (Tl)	2.59/0.83/1.76	Tl⎼P: 17.35% Tl=P: 71.14% Tl≡P: 11.51%
				58.76% (P)		
		π_⊥_ = 1.92	π_⊥_: 0.4064 Tl (sp^99.99^) + 0.9137 P (sp^44.72^)	16.51% (Tl)		
				83.49% (P)		
		π_‖_ = 1.93	π_‖_: 0.4551 Tl (sp^99.99^) + 0.8997 P (sp^94.99^)	14.79% (Tl)		
				85.21% (P)		
R′ = Tbt	2.13	σ = 1.77	σ : 0.6888 Tl (sp^0.94^) + 0.7249 P (sp^38.46^)	47.45% (Tl)	2.08/1.59/0.49	Tl⎼P: 27.42% Tl=P: 63.76% Tl≡P: 8.82%
				52.55% (P)		
		π_⊥_ = 1.94	π_⊥_: 0.4133 Tl (sp^35.51^) + 0.9244 P (sp^87.83^)	23.43% (Tl)		
				82.74% (P)		
		π_‖_ = 1.90	π_‖_: 0.4118 Tl (sp^99.89^) + 0.9077 P (sp^99.99^)	17.28% (Tl)		
				82.72% (P)		
R′ = Ar*	2.20	σ = 1.96	σ: 0.7362 Tl (sp^0.04^) + 0.6767 P (sp^64.96^)	54.20% (Tl)	2.17/1.66/0.51	Tl⎼P: 19.82% Tl=P: 71.69% Tl≡P: 8.49%
				45.80% (P)		
		π_⊥_ = 1.77	π_⊥_: 0.3177 Tl (sp^99.99^) + 0.9482 P (sp^99.99^)	10.09% (Tl)		
				89.91% (P)		
		π_‖_ = 1.92	π_‖_: 0.4083 Tl (sp^99.99^) + 0.9128 P (sp^99.99^)	16.67% (Tl)		
				83.33% (P)		

^(1)^ The value of the Wiberg bond index (WBI) for the Tl–P bond and the occupancy of the corresponding σ and π bonding NBO (see reference [59,60,61]). ^(2)^ NRT; see reference [70,71,72].

## Supplementary Materials

Supplementary materials are available online. The CDA and NRT results concerning the (Si*i*PrDis_2_)Tl≡P(Si*i*PrDis_2_), (Tbt)Tl≡P(Tbt), and (Ar*)Tl≡P(Ar*) molecules are collected in the Supporting Information.
