# Supplementary material for: Substituent Effects on the Stability of Thallium and Phosphorus Triple Bonds: A Density Functional Study
Source: Molecules. 2017 Jul 5;22(7):1111. doi: 10.3390/molecules22071111 (PMC6152323; doi:10.3390/molecules22071111)
Supplement: Supplementary file 1 [file molecules-22-01111-s001.pdf]

Supplementary Materials

Substituent Effects on the Stability of Thallium and Phosphorus Triple Bonds: A Density Functional Study

Jia-Syun Lu,[1] Ming-Chung Yang,[1] and Ming-Der Su[1,2]*

[1]Department of Applied Chemistry, National Chiayi University, Chiayi 60004, Taiwan
[2]Department of Medicinal and Applied Chemistry, Kaohsiung Medical University, Kaohsiung 80708, Taiwan

*E-mail: midesu@mail.ncyu.edu.tw

Table of Contents:

Theoretical Methods S2
References S3
Figure S1 (R′ = Si*i*PrDis$_2$) S4
Figure S2 (R′ = Tbt) S5
Figure S3 (R′ = Ar*) S6
Table S1 (R′ = Si*i*PrDis$_2$) S7
Table S2 (R′ = Tbt) S8
Table S3 (R′ = Ar*) S9
Cartesian Coordinates S10

Theoretical Methods

Using the Gaussian 09 program package,[1] all geometries are fully optimized using hybrid density functional theory at the M06-2X,[2] B3LYP,[3-5] and B3PW91[5,6] levels, in conjunction with the Def2-TZVP[7] and LANL2DZ+dp[8-12] basis sets. These DFT calculations are signified as M06-2X/Def2-TZVP, B3PW91/Def2-TZVP and B3LYP/LANL2DZ+dp, respectively. In order to confirm that the reactants and products have no imaginary frequencies and that the transition states possess only one imaginary frequency, frequency calculations were performed for all structures. Thermodynamic corrections to 298 K, heat capacity corrections and entropy corrections (ΔS) are applied to the three levels of DFT. The relative free energy (ΔG) at 298 K is also computed at the same levels of theory.

Next, Si*i*PrDis$_2$−Tl≡P−Si*i*PrDis$_2$, Tbt−Tl≡P−Tbt, and Ar*−Tl≡P−Ar* are the model reactants for this study. It is known that the B3LYP functional fails to describe nonvalent interactions, such as the London dispersion correctly. As a result, for large ligands, calculations were performed using dispersion-corrected M06-2X method.[2] Because of the limitations of the available memory size and CPU time, frequencies are not computed at the dispersion-corrected M06-2X/Def2-TZVP level of theory for the triply bonded R′Tl≡PR′ systems that have bulky ligands (R′), so the zero-point energies and the Gibbs free energies that are derived using the dispersion-corrected M06-2X/Def2-TZVP cannot be used for these systems.

References:

(1) *Gaussian 09*, version Revision D.01; Frisch, M.J.; Trucks, G.W.; Schlegel, H.B.; Scuseria, G.E.; Robb, M.A.; Cheeseman, J.R.; Scalmani, G.; Barone, V.; Mennucci, B.; Petersson, G.A.; et al. Gaussian, Inc.: Wallingford, CT, USA, 2013.

(2) Zhao, Y.; Truhlar, D.G. Density functionals with broad applicability in chemistry. *Acc. Chem. Res*. **2008**, *41*, 157–167.

(3) Becke, A.D. Density-Functional exchange-energy approximation with correct asymptotic behavior. *Phys. Rev. A* **1988**, *38*, 3098–3100.

(4) Becke, A.D. Density-Functional thermochemistry. *J. Chem. Phys*. **1993**, *98*, 5648–5652.

(5) Lee, C.; Yang, W.; Parr, R.G. Development of the Colle-Salvetti correlation-energy formula into a functional of the electron density. *Phys. Rev. B* **1988**, *37*, 785–789.

(6) Perdew, J.P.; Wang, Y. Accurate and simple analytic representation of the electron-gas correlation energy. *Phys. Rev*. **1992**, *B45*, 13244–13249.

(7) Weigend, F.; Ahlrichs, R. Balanced basis sets of split valence, triple zeta valence and quadruple zeta valence quality for H to Rn: Design and assessment of accuracy. *Phys. Chem. Chem. Phys*. **2005**, *7*, 3297–3305.

(8) Dunning, T.H., Jr.; Hay, P.J. In *Modern Theoretical Chemistry*, Schaefer, H. F., III, Ed.; Plenum: New York, 1976; p1-28.

(9) Hay, P.J.; Wadt, W.R. *Ab initio* effective core potentials for molecular calculations. Potentials for the transition metal atoms Sc to Hg. *J. Chem. Phys*. **1985**, *82*, 270–283.

(10) Hay, P.J.; Wadt, W.R. ***Ab initio* effective core potentials for molecular calculations. Potentials for main group elements Na to Bi.** *J. Chem. Phys*. **1985**, *82*, 284–298.

(11) Hay, P.J.; Wadt, W.R. ***Ab initio* effective core potentials for molecular calculations. Potentials for K to Au including the outermost core orbitals.** *J. Chem. Phys*. **1985**, *82*, 299–310.

(12) Check, C.E.; Faust, T.O.; Bailey, J.M.; Wright, B.J.; Gilbert, T.M.; Sunderlin, L.S. Addition of polarization and diffuse functions to the LANL2DZ basis set for p-block elements. *J. Phys. Chem. A* **2001**, *105*, 8111–8116.

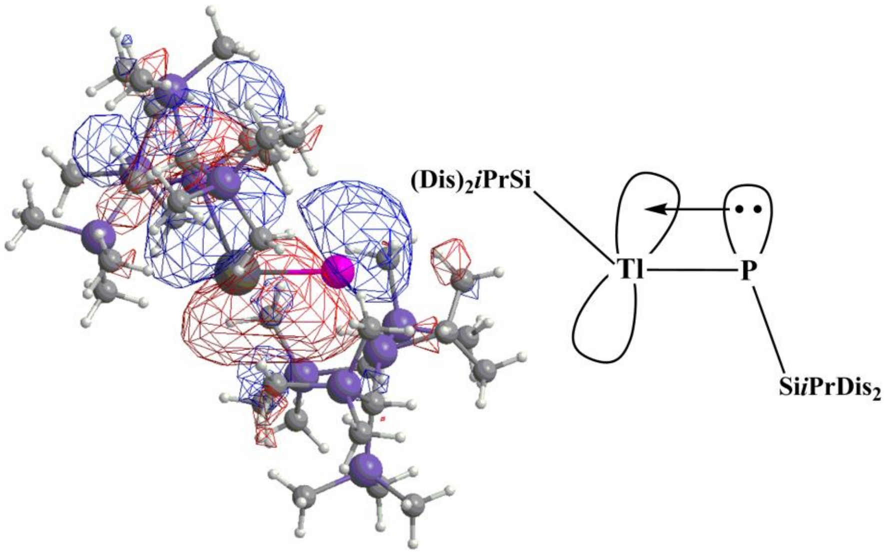

(i) $\pi_{\perp}$

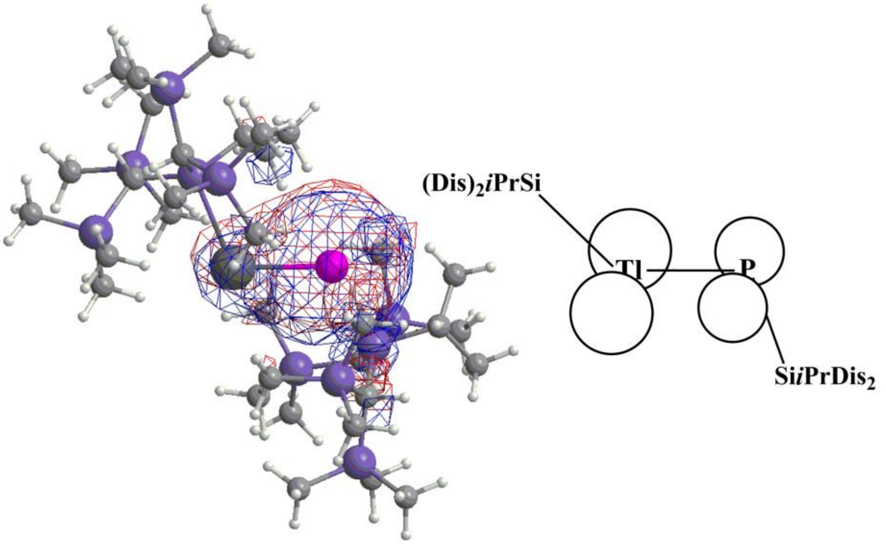

(ii) $\pi_{\parallel}$

Figure S1: The natural Tl≡P π bonding orbitals ((i) and (ii)) of (Si*i*PrDis$_2$)Tl≡P(Si*i*PrDis$_2$)). For comparison, also see Figure 1.

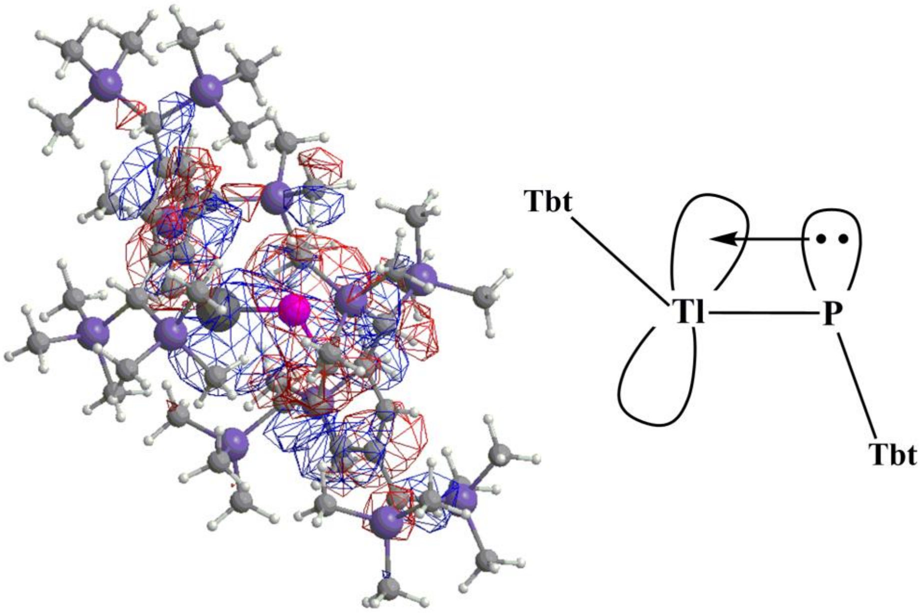

(i) $\pi_{\perp}$

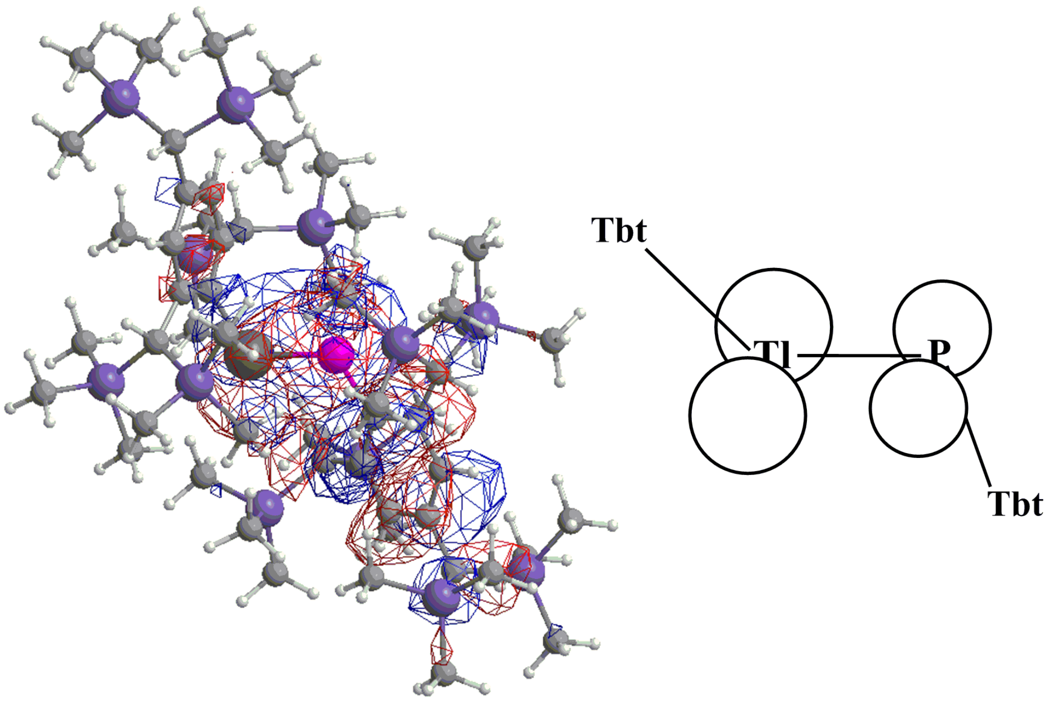

(ii) $\pi_{\parallel}$

Figure S2: The natural Tl≡P π bonding orbitals ((i) and (ii)) of ((Tbt)Tl≡P(Tbt)). For comparison, also see Figure 1.

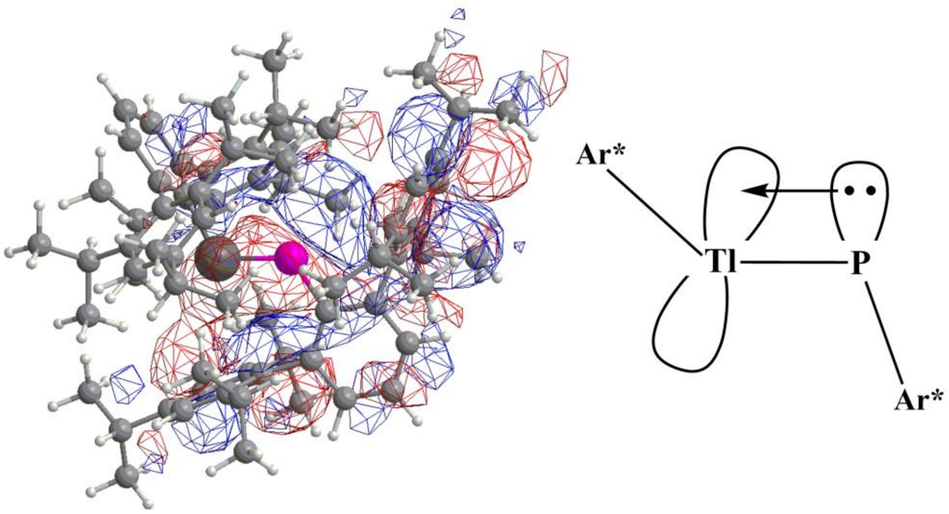

(i) π$_{\perp}$

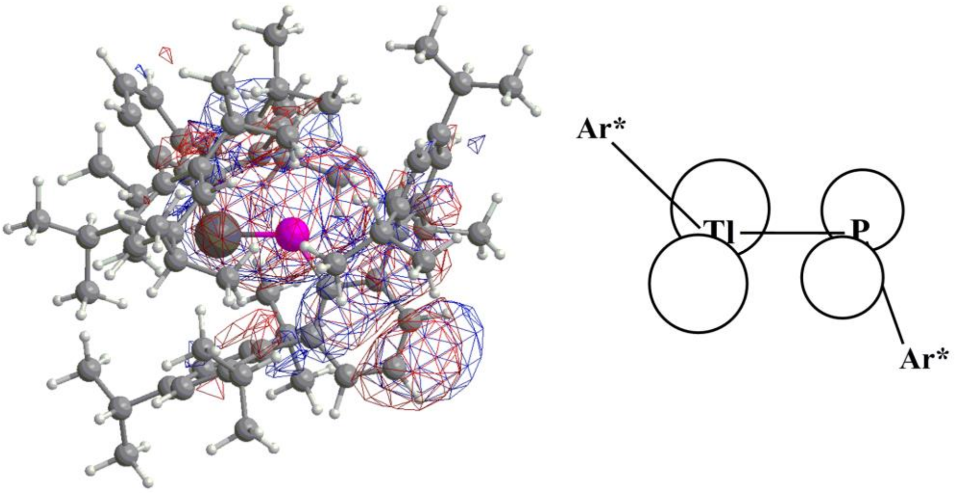

(ii) π$_{\parallel}$

Figure S3: The natural Tl≡P π bonding orbitals ((i) and (ii)) of ((Ar*)Tl≡P(Ar*)). For comparison, also see Figure 1.

Table S1

The charge decomposition analysis (CDA) results[a] for R′Tl≡PR′ (R′ = Si*i*PrDis$_2$) system based on M06-2X orbitals, where X term indicates the number of electrons donated from R′−Tl fragment to R′−P fragment, Y term indicates the number of electrons back donated from R′−P fragment to R′−Tl fragment and W term indicates the number of electrons involved in repulsive polarization. Significant X and Y terms are bolded for easier comparison.

	Orbital	Occupancy	X	Y	X – Y	W
	218	2.000000	-0.000034	-0.000288	0.000524	-0.000721
	219	2.000000	-0.000054	0.001324	-0.001377	-0.007290
	220	2.000000	-0.000072	0.000010	-0.000082	-0.001206
	221	2.000000	0.000158	0.001656	-0.001497	-0.005842
	222	2.000000	0.001052	0.000823	0.000229	-0.004630
	223	2.000000	0.000057	0.000743	-0.000686	-0.002456
	224	2.000000	-0.000198	0.000204	-0.000402	-0.001699
	225	2.000000	0.001883	0.010837	-0.008954	-0.029476
	226	2.000000	-0.003736	0.051334	-0.055070	-0.096576
	227	2.000000	0.037638	0.074490	0.063148	-0.022429
HOMO	228	2.000000	-0.003812	0.062020	-0.065832	-0.010406
LUMO	229	0.000000	0.000000	0.000000	0.000000	0.000000
	230	0.000000	0.000000	0.000000	0.000000	0.000000
sum		352.000000	0.073271	0.279707	-0.206437	-0.187261

[a] For clearness, only list the X, Y, and W terms for HOMO(No.228)−11 ~ LUMO+2. [b] Summation of contributions from all unoccupied and occupied orbitals.

Table S2

The charge decomposition analysis (CDA) results[(a)] for R′Tl≡PR′ (R′ = Tbt) system based on M06-2X orbitals, where X term indicates the number of electrons donated from R′−Tl fragment to R′−P fragment, Y term indicates the number of electrons back donated from R′−P fragment to R′−Tl fragment and W term indicates the number of electrons involved in repulsive polarization. Significant X and Y terms are bolded for easier comparison.

	Orbital	Occupancy	X	Y	X – Y	W
	244	2.000000	-0.000182	0.002656	-0.002837	-0.017427
	245	2.000000	-0.000057	0.001481	-0.001539	-0.005858
	246	2.000000	0.001136	0.000497	0.000639	-0.007476
	247	2.000000	0.002143	0.032910	-0.030768	-0.085173
	248	2.000000	-0.000029	0.001396	-0.001425	-0.002697
	249	2.000000	0.006457	0.016782	-0.010325	-0.012615
	250	2.000000	0.000586	0.000102	0.000484	-0.002452
	251	2.000000	0.006030	0.000462	0.005568	-0.014002
	252	2.000000	-0.000332	0.000616	-0.000947	-0.000091
	253	2.000000	0.054644	0.038525	0.016120	-0.229205
HOMO	254	2.000000	-0.039955	0.045507	-0.085462	0.002804
LUMO	255	0.000000	0.000000	0.000000	0.000000	0.000000
	256	0.000000	0.000000	0.000000	0.000000	0.000000
sum		508.000000	0.111221	0.274270	-0.163049	0.327368

[(a)] For clearness, only list the X, Y, and W terms for HOMO(No.254)−11 ~ LUMO+2. [(b)] Summation of contributions from all unoccupied and occupied orbitals.

Table S3

The charge decomposition analysis (CDA) results[(a)] for R′Tl≡PR′ (R′ = Ar*) system based on M06-2X orbitals, where X term indicates the number of electrons donated from R′−Tl fragment to R′−P fragment, Y term indicates the number of electrons back donated from R′−P fragment to R′−Tl fragment and W term indicates the number of electrons involved in repulsive polarization. Significant X and Y terms are bolded for easier comparison.

	Orbital	Occupancy	X	Y	X – Y	W
	277	2.000000	0.000565	0.000234	0.000331	-0.002550
	278	2.000000	0.001674	0.005633	-0.003959	0.010128
	279	2.000000	0.001757	0.000439	0.001318	-0.010187
	280	2.000000	0.001894	0.001945	-0.000051	-0.005540
	281	2.000000	0.001226	0.005539	-0.004313	-0.006832
	282	2.000000	0.001421	0.001038	0.000384	-0.004852
	283	2.000000	0.002748	0.006598	-0.003849	-0.010218
	284	2.000000	0.003009	0.009053	-0.006044	-0.039804
	285	2.000000	0.000185	0.001824	-0.001639	-0.027482
	286	2.000000	0.024508	0.016964	0.107544	-0.326153
HOMO	287	2.000000	0.001762	0.102084	-0.100322	-0.036265
LUMO	288	0.000000	0.000000	0.000000	0.000000	0.000000
	289	0.000000	0.000000	0.000000	0.000000	0.000000
sum		548.000000	0.186467	0.365639	-0.179172	-0.595339

[(a)] For clearness, only list the X, Y, and W terms for HOMO(No.287)−11 ~ LUMO+2. [(b)] Summation of contributions from all unoccupied and occupied orbitals.

M06-2X/Def2-TZVP

F2Tl-P

Atomic Number	Coordinates (Angstroms) X	Y	Z
P	-0.125054	-2.283317	0.000000
Tl	0.000000	0.105640	0.000000
F	1.618039	1.314363	0.000000
F	-1.409615	1.540401	0.000000

F2Tl-P (TS1)

Atomic Number	Coordinates (Angstroms) X	Y	Z
P	-2.225167	0.274090	0.000000
Tl	0.162036	0.106652	0.000000
F	2.095996	0.555980	0.000000
F	0.154295	-1.972667	0.000000

F-Tl-P-F

Atomic Number	Coordinates (Angstroms) X	Y	Z
P	0.813098	-1.898187	0.000000
Tl	0.000000	0.383688	0.000000
F	-0.687043	2.275755	0.000000
F	-0.668120	-2.565300	0.000000

Tl-PF2 (TS2)

Atomic Number	Coordinates (Angstroms) X	Y	Z
P	2.124422	0.608169	-0.012405
F	3.099827	-0.655473	0.015878
F	-0.835723	1.847964	0.011542
Tl	-0.644979	-0.245123	-0.000749

Tl-PF2

Atomic Number	Coordinates (Angstroms) X	Y	Z
P	1.931507	0.003418	0.603879
F	1.893274	1.215100	-0.505219
F	1.934715	-1.202263	-0.507763
Tl	-0.783019	-0.002059	0.000724

(OH)2Tl-P

Atomic Number	Coordinates (Angstroms) X	Y	Z
P	-0.209463	-2.291507	0.000000
Tl	0.000000	0.088979	0.000000
O	1.609918	1.393151	0.000000
H	2.466572	0.958488	0.000000
O	-1.409358	1.580652	0.000000
H	-0.929104	2.416409	0.000000

(OH)2Tl-P (TS1)

Atomic Number	X	Y	Z
P	2.226677	-0.333083	0.050424
Tl	-0.154095	-0.089258	-0.020503
O	-0.124595	2.022507	-0.055881
H	-0.695345	2.410124	0.613842
O	-2.122145	-0.601446	0.088519
H	-2.249204	-1.552510	0.029415

Coordinates (Angstroms)

HO-Tl-P-OH

Atomic Number	X	Y	Z
P	-1.992414	-0.570251	0.014135
O	-2.593117	0.976375	0.119288
H	-2.828915	1.324911	-0.745695
O	2.395173	0.384913	-0.061981
H	2.820766	0.414330	0.800110
Tl	0.388616	-0.050318	-0.008949

Coordinates (Angstroms)

Tl-P(OH)2 (TS2)

Atomic Number	X	Y	Z
P	2.266255	0.764603	0.034051
Tl	-0.620118	-0.269712	0.003431
O	3.189182	-0.550358	-0.118023
H	2.972150	-1.273105	0.489764
O	-1.384949	1.704207	-0.104312
H	-1.170264	2.419969	0.500205

Coordinates (Angstroms)

Tl-P(OH)2

Atomic Number	Coordinates (Angstroms) X	Y	Z
Tl	0.746208	-0.000035	-0.004138
P	-1.918776	0.000064	-0.604204
O	-1.669095	1.293685	0.474888
H	-2.475064	1.601054	0.899802
O	-1.669686	-1.293516	0.474955
H	-2.475863	-1.600564	0.899690

H2Tl-P

Atomic Number	Coordinates (Angstroms) X	Y	Z
P	0.000457	2.043036	0.000000
Tl	0.000457	-0.347044	0.000000
H	1.439025	-1.298667	0.000000
H	-1.482906	-1.236296	0.000000

H2Tl-P (TS1)

Atomic Number	Coordinates (Angstroms) X	Y	Z
P	2.038126	-0.032569	-0.000053
Tl	-0.350506	-0.003329	0.000009
H	-1.718250	-0.997083	0.000042
H	-0.462630	1.755298	0.000016

H-Tl-P-H

Atomic Number	Coordinates (Angstroms) X	Y	Z
H	1.923564	1.338054	0.000034
P	1.957954	-0.086121	0.000091
Tl	-0.360846	-0.001014	-0.000041
H	-2.064315	0.035923	0.001897

Tl-PH2 (TS2)

Atomic Number	Coordinates (Angstroms) X	Y	Z
Tl	0.418924	-0.018977	0.000758
H	0.071771	1.773996	-0.123760
P	-2.130655	-0.025091	-0.085864
H	-2.044768	0.139524	1.350297

Tl-PH2

Atomic Number	Coordinates (Angstroms) X	Y	Z
Tl	-0.471389	0.000002	-0.000181
P	2.248726	-0.000036	-0.115006
H	2.225974	1.023769	0.869669
H	2.225637	-1.023349	0.870087

(CH3)2Tl-P

Atomic Number	Coordinates (Angstroms) X	Y	Z
Tl	-0.095323	0.000708	-0.000919
P	2.285191	0.003477	0.001734
C	-1.410514	1.785099	0.000050
H	-1.666243	2.007097	1.034362
H	-0.912431	2.639433	-0.448151
H	-2.311160	1.534202	-0.554829
C	-1.396317	-1.795373	0.004505
H	-0.973766	-2.569713	0.638381
H	-1.475818	-2.161312	-1.017042
H	-2.376268	-1.497591	0.368404

(CH3)2Tl-P (TS1)

Atomic Number	Coordinates (Angstroms) X	Y	Z
Tl	-0.111271	-0.056717	-0.000036
P	2.243062	-0.310758	0.000062
C	-0.608811	2.148693	0.000055
H	0.281889	2.768386	-0.006899
H	-1.209472	2.330338	-0.888465
H	-1.197677	2.333406	0.895758
C	-1.989830	-1.174380	-0.000004
H	-1.813198	-2.245353	-0.017119
H	-2.543465	-0.901757	0.895997
H	-2.559170	-0.875469	-0.877564

H3C-Tl-P-CH3

Atomic Number	Coordinates (Angstroms) X	Y	Z
Tl	-0.367718	-0.041483	0.001022
P	1.880573	-0.686087	-0.002230
C	2.719838	1.006056	-0.000380
H	3.786382	0.774221	-0.008396
H	2.513822	1.603122	-0.886419
H	2.525061	1.595442	0.893319
C	-2.500132	0.381398	-0.002520
H	-2.678120	1.318456	-0.526755
H	-2.868531	0.457267	1.017773
H	-3.020302	-0.421814	-0.521446

Tl-P(CH3)2 (TS2)

Atomic Number	Coordinates (Angstroms) X	Y	Z
P	1.864352	0.013192	-0.801195
Tl	-0.596847	-0.242690	0.028588
C	-0.465949	2.158950	0.075409
H	-1.298440	2.360122	0.750483
H	-0.683345	2.483464	-0.942564
H	0.460032	2.590991	0.444789
C	2.674208	-0.089163	0.888057
H	3.166474	-1.074460	0.960496
H	2.003317	0.023766	1.755720
H	3.481755	0.657418	0.952551

Tl-P(CH3)2

Atomic Number	Coordinates (Angstroms) X	Y	Z
Tl	0.789081	-0.000179	0.010664
P	-1.805231	0.000239	-0.751325
C	-2.025461	-1.386696	0.491709
H	-1.308985	-1.384895	1.327968
H	-3.018320	-1.315251	0.939233
H	-1.952437	-2.349726	-0.013329
C	-2.022567	1.387821	0.491492
H	-3.015830	1.318988	0.938550
H	-1.306448	1.384359	1.328140
H	-1.946876	2.350660	-0.013663

(SiH3)2Tl-P

Atomic Number	Coordinates (Angstroms) X	Y	Z
Tl	0.000000	0.000000	0.000000
P	0.000000	0.000000	2.344572
Si	2.081419	0.000000	-1.503844
H	3.323417	0.290228	-0.760895
H	2.189045	-1.332356	-2.134370
H	1.895389	1.020120	-2.557357
Si	-2.097012	-0.033470	-1.484108
H	-3.332764	0.265681	-0.734141
H	-2.205427	-1.376167	-2.092374
H	-1.924543	0.971033	-2.555094

(SiH3)2Tl-P (TS1)

Atomic Coordinates (Angstroms)

Number	X	Y	Z
P	-1.657364	-1.824855	-0.010400
Tl	0.006728	-0.148239	0.004557
Si	-1.190166	2.200471	-0.006720
H	-0.656930	3.008838	-1.131338
H	-2.661916	2.095274	-0.130213
H	-0.853809	2.865821	1.277007
Si	2.567978	0.026341	-0.008625
H	2.995793	0.871145	-1.150191
H	2.998665	0.671215	1.256035
H	3.204320	-1.307486	-0.119566

H3Si-Tl-P-SiH3

Atomic Number	Coordinates (Angstroms) X	Y	Z
Tl	-0.366325	0.104677	-0.006988
P	1.780267	1.006095	0.014213
Si	3.056796	-0.836969	0.003110
H	4.327573	-0.417670	0.644634
H	2.533196	-2.006156	0.754124
H	3.393390	-1.316181	-1.359185
Si	-2.861862	-0.459260	0.016464
H	-3.353559	-0.527518	1.406221
H	-3.587461	0.600884	-0.712216
H	-3.073898	-1.756430	-0.654757

Tl-P(SiH3)2 (TS2)

Atomic Number	Coordinates (Angstroms) X	Y	Z

P	1.451328	0.119094	1.261047
Tl	-0.565523	-0.560233	-0.077485
Si	-1.243530	2.088959	-0.031366
H	-1.871673	2.287795	1.295656
H	-0.174224	3.082189	-0.271747
H	-2.267446	2.226828	-1.095982
Si	2.639920	0.383228	-0.634755
H	3.694214	-0.655288	-0.759010
H	1.819474	0.320463	-1.886724
H	3.287638	1.719871	-0.595900

Tl-P(SiH3)2

Atomic Number	Coordinates (Angstroms) X	Y	Z
Si	2.047802	-1.639890	-0.315894
H	1.437062	-1.567937	-1.678113
H	3.519533	-1.664540	-0.507895
H	1.628397	-2.942115	0.263448
Si	2.044474	1.641665	-0.315461
H	1.630791	2.942815	0.269528
H	3.515180	1.664759	-0.516974
H	1.426201	1.573767	-1.674959
Tl	-1.129500	-0.000375	-0.038178
P	1.402697	-0.000082	1.051759

B3PW91/Def2-TZVP

F2Tl-P

Atomic Number	Coordinates (Angstroms) X	Y	Z
P	-0.032493	-2.288393	0.000000
Tl	0.000000	0.106177	0.000000
F	-1.500322	1.461844	0.000000
F	1.554478	1.396551	0.000000

F2Tl-P (TS1)

Atomic Number	Coordinates (Angstroms) X	Y	Z
P	-1.452334	-1.707987	0.000000
Tl	0.000000	0.193985	0.000000
F	0.687959	2.056459	0.000000
F	1.732598	-0.955681	0.000000

F-Tl-P-F

Atomic Number	Coordinates (Angstroms) X	Y	Z
P	0.802203	-1.900772	0.000000
Tl	0.000000	0.387985	0.000000
F	-0.675233	2.287885	0.000000
F	-0.661772	-2.611800	0.000000

Tl-PF2 (TS2)

Atomic Number	Coordinates (Angstroms) X	Y	Z

P	2.124422	0.608169	-0.012405
F	3.099827	-0.655473	0.015878
F	-0.835723	1.847964	0.011542
Tl	-0.644979	-0.245123	-0.000749

Tl-PF2

Atomic Number	Coordinates (Angstroms) X	Y	Z
P	-1.924893	0.000780	0.600771
F	-2.087473	-1.223939	-0.474499
F	-2.072548	1.229025	-0.473202
Tl	0.818686	-0.000709	-0.005954

(OH)2Tl-P

Atomic Number	Coordinates (Angstroms) X	Y	Z
P	-0.267390	-2.278109	0.000000
Tl	0.000000	0.083106	0.000000
O	1.629612	1.371945	0.000000
H	2.455279	0.875880	0.000000
O	-1.336985	1.643980	0.000000
H	-0.785448	2.436757	0.000000

(OH)2Tl-P (TS1)

Atomic Number	Coordinates (Angstroms) X	Y	Z

P	2.245795	-0.250568	-0.015713
Tl	-0.133660	-0.077991	0.007613
O	-2.031492	-0.824576	-0.016610
H	-1.993810	-1.783713	-0.017090
O	-0.541644	2.001414	-0.116641
H	-0.281578	2.444766	0.702133

HO-Tl-P-OH

Atomic Number	Coordinates (Angstroms) X	Y	Z
P	1.989502	-0.576796	0.013622
O	2.670788	0.935540	-0.119829
H	2.867314	1.304489	0.747731
O	-2.416169	0.350512	0.113620
H	-2.812642	0.372423	-0.765081
Tl	-0.394249	-0.040906	-0.001695

Tl-P(OH)2 (TS2)

Atomic Number	Coordinates (Angstroms) X	Y	Z
P	2.132948	0.745321	0.073152
Tl	-0.600463	-0.272246	0.006741
O	3.070088	-0.555010	-0.165009
H	2.824510	-1.319013	0.376343
O	-1.218251	1.780594	-0.157655
H	-0.995892	2.386458	0.561643

Tl-P(OH)2

Atomic Number	Coordinates (Angstroms) X	Y	Z
Tl	-0.760048	-0.000128	-0.000087
P	1.896949	0.000212	-0.610302
O	1.745489	-1.309688	0.471950
H	2.601392	-1.599536	0.806008
O	1.743221	1.310315	0.471747
H	2.598586	1.601691	0.805983

H2Tl-P

Atomic Number	Coordinates (Angstroms) X	Y	Z
P	-0.000012	-2.004423	0.000000
Tl	-0.000012	0.336815	0.000000
H	-1.375274	1.392827	0.000000
H	1.376423	1.391476	0.000000

H2Tl-P (TS1)

Atomic Number	Coordinates (Angstroms) X	Y	Z
P	2.013036	-0.022276	-0.000052
Tl	-0.342988	-0.005550	0.000009
H	-1.749318	-0.957482	0.000044
H	-0.664193	1.741209	0.000021

H-Tl-P-H

Atomic Number	Coordinates (Angstroms) X	Y	Z
H	1.938079	1.343976	0.000019
P	1.963765	-0.087666	0.000029
Tl	-0.362022	-0.000596	-0.000013
H	-2.070756	0.019258	0.000614

Tl-PH2 (TS2)

Atomic Number	Coordinates (Angstroms) X	Y	Z
Tl	0.418924	-0.018977	0.000758
H	0.071771	1.773996	-0.123760
P	-2.130655	-0.025091	-0.085864
H	-2.044768	0.139524	1.350297

Tl-PH2

Atomic Number	Coordinates (Angstroms) X	Y	Z
Tl	-0.469628	0.000000	-0.000075
P	2.238753	-0.000003	-0.115862
H	2.229322	1.029756	0.872013
H	2.229230	-1.029750	0.872022

(CH3)2Tl-P

Atomic	Coordinates (Angstroms)

Number	X	Y	Z
C	-1.504422	1.704714	0.000063
H	-1.982662	1.713120	0.978169
H	-0.981415	2.638601	-0.186926
H	-2.234734	1.498595	-0.779806
C	-1.524369	-1.688287	0.001778
H	-1.001135	-2.630390	0.141683
H	-2.035777	-1.664881	-0.959173
H	-2.226799	-1.503167	0.812118
P	2.268639	-0.002818	0.000758
Tl	-0.066597	-0.001336	-0.000352

(CH3)2Tl-P (TS1)

Atomic Number	Coordinates (Angstroms) X	Y	Z
Tl	-0.111271	-0.056717	-0.000036
P	2.243062	-0.310759	0.000062
C	-0.608811	2.148693	0.000055
H	0.281890	2.768386	-0.006899
H	-1.209472	2.330339	-0.888465
H	-1.197677	2.333407	0.895758
C	-1.989831	-1.174379	-0.000004
H	-1.813199	-2.245352	-0.017119
H	-2.543466	-0.901756	0.895997
H	-2.559171	-0.875468	-0.877564

H3C-Tl-P-CH3

Atomic Number	Coordinates (Angstroms) X	Y	Z

Tl	-0.373189	-0.035902	0.000753
P	1.884079	-0.684844	-0.001558
C	2.775696	0.975281	-0.000272
H	3.836718	0.710696	-0.008621
H	2.588383	1.580137	-0.887609
H	2.600478	1.573022	0.894279
C	-2.518164	0.361540	-0.001603
H	-2.676007	1.393280	-0.316404
H	-2.922291	0.214122	0.998852
H	-3.005341	-0.311470	-0.706854

Tl-P(CH3)2 (TS2)

Atomic Number	Coordinates (Angstroms) X	Y	Z
P	1.864352	0.013192	-0.801195
Tl	-0.596847	-0.242690	0.028588
C	-0.465949	2.158950	0.075409
H	-1.298440	2.360122	0.750483
H	-0.683345	2.483464	-0.942564
H	0.460032	2.590991	0.444789
C	2.674208	-0.089163	0.888057
H	3.166474	-1.074460	0.960496
H	2.003317	0.023766	1.755720
H	3.481755	0.657418	0.952551

Tl-P(CH3)2

Atomic Number	Coordinates (Angstroms) X	Y	Z

Tl	-0.864923	-0.000004	0.022847
P	1.759638	0.000002	-0.654764
C	2.377441	1.428788	0.377587
H	2.069523	1.399655	1.427072
H	3.470913	1.450488	0.351872
H	2.026649	2.367154	-0.059017
C	2.377507	-1.428734	0.377585
H	3.470976	-1.450671	0.351513
H	2.070037	-1.399541	1.427217
H	2.026417	-2.367102	-0.058794

(SiH3)2Tl-P

Atomic Number	Coordinates (Angstroms) X	Y	Z
Tl	0.018472	0.132859	0.005622
P	0.371054	2.431595	-0.011181
Si	-2.296102	-1.026641	-0.006495
H	-3.366238	-0.034670	0.273443
H	-2.513164	-1.626533	-1.350707
H	-2.329388	-2.097579	1.023536
Si	1.895789	-1.651097	-0.006457
H	3.211165	-1.012679	0.258004
H	1.917899	-2.299863	-1.345443
H	1.622046	-2.675815	1.034789

(SiH3)2Tl-P (TS1)

Atomic Number	Coordinates (Angstroms) X	Y	Z
P	-1.657364	-1.824855	-0.010400

Tl	0.006728	-0.148239	0.004557
Si	-1.190166	2.200471	-0.006720
H	-0.656930	3.008838	-1.131338
H	-2.661916	2.095274	-0.130213
H	-0.853809	2.865821	1.277007
Si	2.567978	0.026341	-0.008625
H	2.995793	0.871145	-1.150191
H	2.998665	0.671215	1.256035
H	3.204320	-1.307486	-0.119566

H3Si-Tl-P-SiH3

Atomic Number	Coordinates (Angstroms) X	Y	Z
P	1.846472	-1.021866	0.006275
Tl	-0.329704	-0.172337	-0.002575
Si	2.761370	1.029502	-0.000032
H	3.409987	1.349830	-1.304335
H	1.817879	2.160776	0.283548
H	3.826952	1.092464	1.044495
Si	-2.781903	0.581078	0.006930
H	-3.598845	-0.555859	-0.492956
H	-3.216181	0.942202	1.380869
H	-2.943417	1.749775	-0.893757

Tl-P(SiH3)2 (TS2)

Atomic Number	Coordinates (Angstroms) X	Y	Z
P	1.451328	0.119094	1.261047
Tl	-0.565523	-0.560233	-0.077485

Si	-1.243530	2.088959	-0.031366
H	-1.871673	2.287795	1.295656
H	-0.174225	3.082189	-0.271747
H	-2.267446	2.226827	-1.095982
Si	2.639920	0.383228	-0.634755
H	3.694214	-0.655287	-0.759010
H	1.819474	0.320463	-1.886724
H	3.287638	1.719871	-0.595900

Tl-P(SiH3)2

Atomic Number	Coordinates (Angstroms) X	Y	Z
Si	1.765209	1.674480	0.359493
H	0.562118	1.850182	1.268143
H	2.917453	1.507342	1.286797
H	1.889756	2.974533	-0.352249
Si	1.906588	-1.603651	0.365862
H	2.052506	-2.901772	-0.347807
H	3.115034	-1.365303	1.199547
H	0.784198	-1.826271	1.356090
Tl	-1.049365	-0.018672	0.023921
P	1.484821	0.018808	-1.100205

B3LYP/LANL2DZ+dp

F2Tl-P

Atomic Number	Coordinates (Angstroms) X	Y	Z

P	-0.000961	-2.308891	0.000000
Tl	0.000000	0.156777	0.000000
F	-1.534787	1.219987	0.000000
F	1.536388	1.217171	0.000000

F2Tl-P (TS1)

Atomic Number	Coordinates (Angstroms) X	Y	Z
P	-1.092174	-1.947070	0.000000
Tl	0.000000	0.218061	-0.000000
F	0.167432	2.055641	-0.000000
F	1.652857	-0.773074	0.000000

F-Tl-P-F

Atomic Number	Coordinates (Angstroms) X	Y	Z
P	1.998273	-0.578570	0.000000
Tl	-0.400293	-0.052085	0.000000
F	-2.210168	0.387439	0.000000
F	2.482354	1.045606	0.000000

Tl-PF2 (TS2)

Atomic Number	Coordinates (Angstroms) X	Y	Z
P	2.139712	0.669539	-0.084891
F	3.024958	-0.715144	0.114544
F	-0.912718	1.693764	0.094948

Tl	-0.630936	-0.232724	-0.007556

Tl-PF2

Atomic Number	Coordinates (Angstroms) X	Y	Z
P	1.941858	0.025334	0.616527
F	1.726741	1.283282	-0.537536
F	1.916365	-1.235133	-0.537803
Tl	-0.764393	-0.010041	0.005310

(OH)2Tl-P

Atomic Number	Coordinates (Angstroms) X	Y	Z
P	-0.103621	-2.322015	0.000000
Tl	0.000000	0.140003	0.000000
O	1.603126	1.258883	0.000000
H	2.454993	0.799917	0.000000
O	-1.553229	1.298123	0.000000
H	-1.299860	2.234043	0.000000

(OH)2Tl-P (TS1)

Atomic Number	Coordinates (Angstroms) X	Y	Z
P	2.179746	-0.503303	0.003230
Tl	-0.209177	-0.071718	0.000332
O	-2.107681	-0.324235	0.009686

H	-2.405030	-1.244916	-0.000660
O	0.372383	1.860061	-0.110288
H	0.534569	2.316995	0.730150

HO-Tl-P-OH

Atomic	Coordinates (Angstroms)		
Number	X	Y	Z
P	2.006117	-0.602985	-0.010915
O	2.679881	0.967567	-0.118559
H	2.913943	1.296636	0.762253
O	-2.314421	0.350394	0.068995
H	-2.777675	0.436436	-0.778533
Tl	-0.409280	-0.039901	0.007118

Tl-P(OH)2 (TS2)

Atomic	Coordinates (Angstroms)		
Number	X	Y	Z
P	-2.234791	0.715135	-0.085696
Tl	0.644642	-0.252635	-0.007841
O	-3.216052	-0.600840	0.177607
H	-2.974049	-1.376970	-0.360030
O	1.128006	1.687686	0.164638
H	0.984295	2.418598	-0.457330

Tl-P(OH)2

Atomic	Coordinates (Angstroms)		
Number	X	Y	Z

Tl	-0.739495	-0.000374	-0.005052
P	1.910138	0.000897	-0.620734
O	1.647894	-1.335823	0.493790
H	2.469336	-1.639322	0.910674
O	1.641641	1.337319	0.493503
H	2.461445	1.644192	0.911239

H2Tl-P

Atomic Number	X	Y	Z
	Coordinates (Angstroms)		
P	0.000005	-2.066759	0.000000
Tl	0.000005	0.353239	0.000000
H	1.495330	1.194944	0.000000
H	-1.495857	1.194063	0.000000

H2Tl-P (TS1)

Atomic Number	X	Y	Z
	Coordinates (Angstroms)		
P	2.038126	-0.032569	-0.000053
Tl	-0.350506	-0.003329	0.000009
H	-1.718250	-0.997083	0.000042
H	-0.462630	1.755298	0.000016

H-Tl-P-H

Atomic Number	X	Y	Z
	Coordinates (Angstroms)		

H	1.930018	1.360079	0.000007
P	1.967225	-0.088656	0.000009
Tl	-0.362540	-0.000517	-0.000004
H	-2.072672	0.011605	0.000186

Tl-PH2 (TS2)

Atomic Number	Coordinates (Angstroms) X	Y	Z
Tl	0.418924	-0.018977	0.000758
H	0.071771	1.773997	-0.123760
P	-2.130654	-0.025091	-0.085864
H	-2.044768	0.139524	1.350298

Tl-PH2

Atomic Number	Coordinates (Angstroms) X	Y	Z
Tl	-0.469254	0.000000	-0.000135
P	2.237830	-0.000002	-0.116938
H	2.221101	1.043670	0.882494
H	2.221034	-1.043662	0.882505

(CH3)2Tl-P

Atomic Number	Coordinates (Angstroms) X	Y	Z
C	-1.298776	1.837662	-0.000763

H	-1.856648	1.887774	0.942645
H	-0.661818	2.721932	-0.100994
H	-2.007685	1.791541	-0.836498
C	-1.295911	-1.839407	0.000346
H	-0.657244	-2.724181	0.084182
H	-1.867088	-1.881985	-0.935401
H	-1.993291	-1.801970	0.846204
P	2.296955	0.000750	-0.000096
Tl	-0.121512	0.000075	0.000047

(CH3)2Tl-P (TS1)

Atomic Number	Coordinates (Angstroms) X	Y	Z
Tl	-0.152936	-0.047971	0.000041
P	2.172277	-0.577835	-0.000106
C	0.012631	2.186668	-0.000082
H	1.042266	2.553905	-0.006308
H	-0.516094	2.529083	-0.897972
H	-0.505120	2.527923	0.904247
C	-2.139824	-0.865571	-0.000232
H	-2.112113	-1.959939	-0.014776
H	-2.665648	-0.523883	0.899687
H	-2.676450	-0.500461	-0.884724

H3C-Tl-P-CH3

Atomic Number	Coordinates (Angstroms) X	Y	Z
Tl	-0.378176	-0.019250	0.000052
P	1.876000	-0.716940	-0.000090

C	2.828562	0.948425	-0.000045
H	3.888468	0.656360	0.000738
H	2.657093	1.556112	-0.896304
H	2.656010	1.556843	0.895505
C	-2.509754	0.301979	-0.000026
H	-2.801383	0.886552	-0.880750
H	-2.811222	0.830167	0.912357
H	-3.009528	-0.675095	-0.033976

Tl-P(CH3)2 (TS2)

Atomic Number	Coordinates (Angstroms) X	Y	Z
P	1.864352	0.013192	-0.801195
Tl	-0.596847	-0.242690	0.028588
C	-0.465949	2.158950	0.075409
H	-1.298440	2.360122	0.750483
H	-0.683345	2.483464	-0.942564
H	0.460032	2.590991	0.444789
C	2.674208	-0.089163	0.888057
H	3.166474	-1.074460	0.960496
H	2.003317	0.023766	1.755720
H	3.481755	0.657418	0.952551

Tl-P(CH3)2

Atomic Number	Coordinates (Angstroms) X	Y	Z
Tl	0.836277	-0.000038	0.020530
P	-1.769324	-0.000003	-0.719955
C	-2.249394	-1.438699	0.433017

H	-1.767089	-1.417005	1.424595
H	-3.336542	-1.424816	0.597298
H	-2.002517	-2.393869	-0.051354
C	-2.248736	1.439019	0.432925
H	-3.335959	1.426042	0.596777
H	-1.766832	1.416796	1.424669
H	-2.000871	2.394017	-0.051269

(SiH3)2Tl-P

Atomic Number	Coordinates (Angstroms) X	Y	Z
Tl	0.000211	0.082562	-0.006308
P	-0.027101	2.483196	0.011461
Si	2.242488	-1.225670	0.007819
H	3.380526	-0.363097	-0.391463
H	2.481693	-1.739944	1.380104
H	2.151867	-2.374024	-0.928237
Si	-2.220405	-1.263317	0.007808
H	-3.375120	-0.417524	-0.379448
H	-2.444163	-1.792490	1.377075
H	-2.114563	-2.402584	-0.937752

(SiH3)2Tl-P (TS1)

Atomic Number	Coordinates (Angstroms) X	Y	Z
P	1.657364	1.824855	-0.010400
Tl	-0.006728	0.148239	0.004557
Si	1.190167	-2.200471	-0.006720
H	0.656931	-3.008838	-1.131338
H	2.661917	-2.095273	-0.130213

H	0.853810	-2.865821	1.277007
Si	-2.567978	-0.026342	-0.008625
H	-2.995793	-0.871146	-1.150191
H	-2.998665	-0.671216	1.256035
H	-3.204320	1.307485	-0.119566

H3Si-Tl-P-SiH3

Atomic Number	Coordinates (Angstroms) X	Y	Z
Tl	-0.322306	-0.205196	-0.001275
P	1.871006	-1.011481	0.003961
Si	2.683153	1.107485	0.000140
H	3.550705	1.338683	-1.184598
H	1.634934	2.174623	-0.032058
H	3.500295	1.364693	1.215171
Si	-2.754762	0.644824	0.003212
H	-2.958104	1.542937	-1.156993
H	-3.647201	-0.533567	-0.106604
H	-3.036392	1.373348	1.262041

Tl-P(SiH3)2 (TS2)

Atomic Number	Coordinates (Angstroms) X	Y	Z
P	-1.451328	-0.119094	1.261047
Tl	0.565523	0.560233	-0.077485
Si	1.243530	-2.088959	-0.031366
H	1.871672	-2.287795	1.295656
H	0.174223	-3.082189	-0.271747
H	2.267446	-2.226829	-1.095982

Si	-2.639920	-0.383228	-0.634755
H	-3.694214	0.655289	-0.759010
H	-1.819474	-0.320463	-1.886724
H	-3.287638	-1.719870	-0.595900

Tl-P(SiH3)2

Atomic Number	Coordinates (Angstroms) X	Y	Z
Si	1.904427	-1.677822	-0.349088
H	0.789438	-1.914792	-1.335205
H	3.122588	-1.455537	-1.167011
H	2.039874	-2.947730	0.406793
Si	1.894859	1.682655	-0.348711
H	2.028208	2.952565	0.406939
H	3.109874	1.465620	-1.172349
H	0.774633	1.915810	-1.329678
Tl	-1.073266	-0.001257	-0.028838
P	1.458664	0.001213	1.086374

B97D3/LANL2DZ+dp

(SiMe(SitBu3)2)2Tl-P

Atomic Number	Coordinates (Angstroms) X	Y	Z
Tl	-0.111411	1.034668	1.299947
P	0.066880	2.455329	-0.714405
Si	-2.762988	0.402183	2.037327
Si	2.471689	0.504525	2.276967

Si	-4.217304	2.360055	1.142313
Si	-3.249370	-2.153569	1.807597
Si	3.943415	-0.142796	0.208067
Si	2.888791	2.154325	4.224712
C	-2.703898	0.680293	3.946639
H	-2.010871	1.484620	4.210170
H	-2.361458	-0.223450	4.464686
H	-3.685642	0.947642	4.359435
C	2.275428	-1.173074	3.204382
H	3.217375	-1.504087	3.664978
H	1.949585	-1.968101	2.523858
H	1.526109	-1.101748	4.002237
C	5.699356	-0.785497	0.823204
C	3.024808	-1.600691	-0.765935
C	4.178991	1.388722	-1.004409
C	5.314754	1.198791	-2.043512
H	6.304117	1.160941	-1.566416
H	5.318358	2.065316	-2.731018
H	5.194142	0.294829	-2.654367
C	2.891643	1.670845	-1.796701
H	2.629856	0.892467	-2.526873
H	2.968853	2.630632	-2.334949
H	2.007887	1.753253	-1.118485
C	4.483249	2.677502	-0.220412
H	5.438271	2.630696	0.314322
H	3.691207	2.894341	0.504197
H	4.535862	3.533498	-0.918445
C	3.621245	-1.829756	-2.179746
H	4.700293	-2.030838	-2.165474
H	3.439073	-0.982778	-2.854740
H	3.129014	-2.711723	-2.631694
C	1.522749	-1.312225	-0.959147
H	1.318150	-0.417912	-1.558582
H	1.016380	-1.181340	0.004247
H	1.043771	-2.171052	-1.464844

C	3.134439	-2.950459	-0.015700
H	4.160252	-3.344095	-0.018039
H	2.498950	-3.699148	-0.524196
H	2.797040	-2.888393	1.026622
C	6.495601	-1.549332	-0.264903
H	7.505017	-1.774241	0.128157
H	6.625794	-0.974577	-1.190939
H	6.029803	-2.511374	-0.520542
C	6.567983	0.416032	1.266601
H	7.456170	0.050951	1.814769
H	6.031306	1.100861	1.932831
H	6.930320	0.997076	0.406789
C	5.572284	-1.753443	2.023636
H	6.583102	-2.028295	2.378525
H	5.049400	-2.682275	1.762302
H	5.040532	-1.307264	2.869657
C	3.325933	3.926665	3.517907
C	1.231258	2.265763	5.292735
C	4.335416	1.477652	5.392053
C	4.777931	3.933148	2.980205
H	4.996027	3.073201	2.337856
H	5.514017	3.940668	3.797310
H	4.947323	4.844130	2.377206
C	2.368642	4.296193	2.357159
H	2.253073	3.493160	1.617535
H	2.753719	5.184997	1.824833
H	1.360884	4.543195	2.713292
C	3.239781	5.068760	4.562787
H	3.897307	4.903669	5.427730
H	2.218206	5.225599	4.935868
H	3.557390	6.014560	4.084611
C	5.543817	1.008397	4.560658
H	6.309914	0.571256	5.228449
H	6.020424	1.828470	4.010317
H	5.261922	0.235321	3.839269

C	3.875913	0.255668	6.225811
H	4.745364	-0.149855	6.776558
H	3.479138	-0.554137	5.598242
H	3.113368	0.511961	6.973828
C	4.868461	2.547605	6.377637
H	5.630853	2.080852	7.029652
H	4.092088	2.967960	7.028450
H	5.360029	3.377560	5.851793
C	1.491447	2.869158	6.697953
H	2.095787	2.208253	7.332783
H	0.521314	3.011685	7.210967
H	1.986485	3.849336	6.659134
C	0.176398	3.166750	4.618137
H	-0.060632	2.843744	3.592297
H	0.483144	4.219482	4.573968
H	-0.758308	3.129652	5.204959
C	0.566797	0.886076	5.495033
H	1.231023	0.154368	5.971620
H	0.223593	0.466873	4.539934
H	-0.322868	0.999076	6.141230
C	-3.610187	4.058315	1.956499
C	-2.319339	4.599816	1.302194
H	-2.004589	5.516964	1.833933
H	-2.448387	4.870186	0.245601
H	-1.485209	3.887430	1.358288
C	-4.691359	5.164100	1.836426
H	-4.263987	6.112859	2.211761
H	-5.578433	4.949408	2.447776
H	-5.022031	5.342144	0.805408
C	-3.285482	3.912324	3.460384
H	-3.082010	4.913857	3.883219
H	-2.384522	3.310973	3.609414
H	-4.095999	3.464015	4.048244
C	-4.114205	2.518599	-0.818397
C	-6.079872	2.015292	1.679627

C	-2.669246	2.331504	-1.331954
H	-1.973019	3.080760	-0.866618
H	-2.614147	2.500152	-2.421947
H	-2.301980	1.315943	-1.138423
C	-4.636633	3.875616	-1.349778
H	-4.022940	4.723897	-1.020397
H	-5.676533	4.070918	-1.054415
H	-4.605728	3.860916	-2.455008
C	-4.918117	1.408130	-1.532992
H	-4.591162	0.407907	-1.228562
H	-4.760623	1.487443	-2.624408
H	-5.998555	1.491530	-1.354650
C	-7.124730	2.912153	0.970677
H	-6.966086	3.984140	1.143808
H	-8.131366	2.661676	1.355945
H	-7.146247	2.742298	-0.115131
C	-6.453591	0.553691	1.361316
H	-5.674019	-0.141325	1.693763
H	-6.611775	0.387814	0.288286
H	-7.391713	0.279333	1.877389
C	-6.259943	2.204717	3.204993
H	-6.144249	3.251281	3.517335
H	-5.555425	1.597140	3.790150
H	-7.280192	1.888458	3.491428
C	-3.492106	-2.557201	-0.106668
C	-4.798376	-2.679996	2.963663
C	-1.635472	-3.107170	2.456145
C	-6.111313	-2.907027	2.177891
H	-6.430957	-2.043317	1.587821
H	-6.918708	-3.130035	2.900040
H	-6.031019	-3.774207	1.505226
C	-4.567353	-4.001014	3.734791
H	-3.724551	-3.939111	4.435099
H	-4.409502	-4.858600	3.066675
H	-5.471280	-4.213492	4.335253

C	-5.060498	-1.607134	4.045031
H	-4.203950	-1.498016	4.724968
H	-5.931585	-1.908315	4.656423
H	-5.282274	-0.621243	3.621954
C	-4.893224	-2.175561	-0.642437
H	-5.234128	-1.199201	-0.283910
H	-5.655322	-2.916624	-0.373781
H	-4.857852	-2.131192	-1.746241
C	-3.300668	-4.059853	-0.429228
H	-2.272182	-4.407884	-0.260960
H	-3.533860	-4.234251	-1.496498
H	-3.980219	-4.696967	0.160566
C	-2.467279	-1.743215	-0.935657
H	-1.425760	-1.974026	-0.691308
H	-2.601710	-0.661564	-0.796893
H	-2.611076	-1.957214	-2.011096
C	-1.778478	-4.656253	2.575265
H	-0.769502	-5.106151	2.527164
H	-2.371955	-5.111652	1.772954
H	-2.214398	-4.963926	3.531605
C	-0.454650	-2.873248	1.497830
H	0.459887	-3.309238	1.934720
H	-0.267395	-1.804608	1.338327
H	-0.597242	-3.350209	0.520001
C	-1.192011	-2.575447	3.838847
H	-0.344553	-3.180293	4.210023
H	-1.991608	-2.627719	4.593139
H	-0.843650	-1.535807	3.777703

SiMe(SitBu3)2-Tl-P-SiMe(SitBu3)2

Atomic Number	Coordinates (Angstroms) X	Y	Z

Tl	-0.026091	-0.089952	1.686944
P	0.679914	0.886296	-0.372918
Si	2.364034	-0.017630	-1.645058
Si	-1.262896	-0.768825	3.950343
C	-0.373465	-2.478794	4.147344
H	0.625331	-2.480705	3.688438
H	-0.950554	-3.276000	3.665616
H	-0.243412	-2.753756	5.201915
Si	4.713419	0.012644	-0.330516
Si	-0.268720	0.772477	5.665825
Si	-3.675817	-1.356086	3.471240
C	6.327784	-0.104245	-1.480543
C	4.801853	1.768772	0.631679
C	4.755665	-1.458205	0.999371
C	6.585431	-1.537228	-2.000380
H	7.434144	-1.513135	-2.709604
H	5.726256	-1.959071	-2.531002
H	6.862930	-2.224825	-1.189106
C	7.606963	0.301610	-0.698782
H	8.479772	0.158838	-1.363966
H	7.775129	-0.312947	0.193981
H	7.607428	1.354786	-0.393636
C	6.246711	0.825350	-2.714714
H	7.192858	0.747565	-3.283299
H	6.111136	1.879331	-2.446877
H	5.434724	0.549427	-3.395446
C	5.336342	2.929591	-0.243640
H	6.404367	2.835628	-0.479863
H	5.212364	3.873246	0.320114
H	4.783551	3.044972	-1.182836
C	5.725703	1.702616	1.876338
H	6.749586	1.379672	1.646601
H	5.322027	1.048618	2.661074
H	5.788167	2.718905	2.308341
C	3.424292	2.225751	1.146119

H	2.917381	1.456522	1.736555
H	2.742746	2.488421	0.330759
H	3.552348	3.117403	1.789415
C	6.102870	-1.598266	1.757713
H	6.896921	-1.978461	1.098575
H	5.972639	-2.346551	2.562590
H	6.460062	-0.675379	2.225797
C	4.496201	-2.852744	0.391411
H	5.227939	-3.130892	-0.376171
H	3.495611	-2.928779	-0.037002
H	4.562774	-3.609633	1.196305
C	3.634796	-1.232711	2.038878
H	3.784709	-0.329121	2.640046
H	3.594164	-2.092168	2.733705
H	2.652259	-1.151343	1.546585
C	2.676204	-2.375123	-3.897464
C	2.365986	-3.251693	-5.149814
H	3.302304	-3.751613	-5.452218
H	2.030876	-2.662999	-6.011399
H	1.625268	-4.035736	-4.959731
C	3.772866	-1.376733	-4.360911
H	4.704558	-1.930037	-4.554875
H	3.989609	-0.637252	-3.581903
H	3.534249	-0.836024	-5.279120
C	3.437418	-3.302915	-2.924735
H	4.354173	-3.662127	-3.417894
H	2.874311	-4.192704	-2.628517
H	3.737912	-2.758468	-2.026590
C	0.479791	-0.827528	-4.270147
C	-0.476886	-1.728764	-5.118322
H	0.036982	-2.517159	-5.678733
H	-0.975904	-1.075670	-5.854439
H	-1.269681	-2.192691	-4.521219
C	-0.465776	0.247617	-3.646892
H	-0.017737	0.805952	-2.819457

H	-1.404365	-0.171800	-3.273895
H	-0.732925	0.979232	-4.426692
C	1.298936	-0.055125	-5.344454
H	1.764776	-0.721931	-6.080002
H	2.076409	0.591512	-4.925167
H	0.608989	0.595191	-5.903803
C	0.512794	-2.654290	-2.256797
C	-0.866252	-2.074980	-1.846687
H	-0.782471	-1.074481	-1.412675
H	-1.290007	-2.735445	-1.075966
H	-1.589431	-2.055871	-2.668865
C	0.182444	-4.036551	-2.899749
H	1.061782	-4.679432	-3.028191
H	-0.323062	-3.948325	-3.867158
H	-0.504130	-4.567874	-2.218546
C	1.180677	-2.995036	-0.898600
H	2.022597	-3.682178	-0.992642
H	0.443421	-3.490211	-0.246032
H	1.520712	-2.092268	-0.375105
C	-3.695580	-2.227153	1.682119
C	-3.431316	-1.208675	0.548654
H	-2.539776	-0.585740	0.712380
H	-4.287089	-0.539012	0.390588
H	-3.271260	-1.753744	-0.395661
C	-2.601673	-3.316417	1.582148
H	-1.589250	-2.887042	1.595751
H	-2.709210	-3.856775	0.624456
H	-2.668679	-4.062793	2.389444
C	-5.045278	-2.911403	1.302372
H	-5.138470	-3.918325	1.722849
H	-5.079095	-3.022629	0.203346
H	-5.933545	-2.339010	1.597395
C	-4.751879	0.289172	3.368286
C	-3.990321	1.362655	2.550879
H	-3.015820	1.598006	3.000707

H	-4.577689	2.298993	2.540297
H	-3.815011	1.074684	1.508898
C	-6.122520	0.043122	2.692262
H	-6.724414	0.969435	2.744133
H	-6.697538	-0.748599	3.199372
H	-6.031972	-0.226325	1.631269
C	-5.049668	0.918407	4.751908
H	-4.182763	0.905789	5.419190
H	-5.875571	0.415230	5.267886
H	-5.348546	1.972871	4.613128
C	-4.326107	-2.646608	4.860603
C	-3.150659	-3.312283	5.612449
H	-2.502780	-2.590158	6.125922
H	-2.524553	-3.916772	4.942386
H	-3.555750	-3.991581	6.385061
C	-5.156799	-3.811117	4.270231
H	-6.059741	-3.465346	3.748401
H	-5.486316	-4.459857	5.102886
H	-4.572566	-4.439400	3.584974
C	-5.245536	-1.994771	5.919913
H	-4.775332	-1.169861	6.465886
H	-5.519432	-2.765923	6.663525
H	-6.181931	-1.628483	5.474459
C	-0.670128	2.648440	5.232810
C	-1.004449	0.267336	7.418013
C	1.697527	0.540297	5.698420
C	-2.140032	3.004330	5.543905
H	-2.340134	4.040303	5.213570
H	-2.365799	2.957029	6.618614
H	-2.843295	2.352931	5.015676
C	0.204004	3.642292	6.040775
H	1.270599	3.575148	5.792158
H	0.098586	3.509530	7.126537
H	-0.119542	4.672858	5.803805
C	-0.451243	2.948745	3.729591

H	0.571967	2.752042	3.387851
H	-0.661217	4.016498	3.535600
H	-1.138536	2.372097	3.092096
C	-2.531159	0.086808	7.319394
H	-3.054331	1.039444	7.173365
H	-2.920185	-0.364444	8.250724
H	-2.799690	-0.578165	6.490069
C	-0.743849	1.308636	8.533654
H	0.322399	1.502688	8.704998
H	-1.167623	0.929977	9.482873
H	-1.235906	2.268852	8.323484
C	-0.425583	-1.088894	7.890559
H	0.651886	-1.041667	8.096113
H	-0.595781	-1.895576	7.162440
H	-0.927146	-1.387425	8.829495
C	2.142703	-0.934668	5.564198
H	3.229771	-1.001711	5.754879
H	1.975313	-1.306587	4.547284
H	1.640774	-1.613176	6.265023
C	2.382915	1.288702	4.536886
H	3.462986	1.058155	4.541641
H	2.287884	2.379430	4.600048
H	1.993358	0.975054	3.557963
C	2.312165	1.081194	7.016371
H	3.414015	1.041125	6.929955
H	2.038697	0.471820	7.888502
H	2.040070	2.123048	7.228780
C	2.551762	1.651307	-2.616142
H	3.282459	1.534046	-3.388950
H	1.611593	1.918194	-3.051749
H	2.864775	2.422177	-1.943325
Si	1.149414	-1.517257	-3.062849

Tl-P(SiMe(SitBu3)2)2

Atomic Number	Coordinates (Angstroms) X	Y	Z
P	-0.120086	0.965394	1.383385
Tl	-0.210261	1.497708	-1.248262
Si	-2.364790	0.253136	1.881986
Si	2.043199	0.514059	2.358133
Si	-4.137767	2.391773	1.346627
Si	-2.966788	-2.225178	1.299816
Si	3.956536	-0.162415	0.426667
Si	2.522678	2.252087	4.266830
C	-2.493577	0.299574	3.783992
H	-2.197704	1.290662	4.145045
H	-1.828374	-0.435507	4.249937
H	-3.512215	0.093160	4.138272
C	1.969878	-1.128720	3.329516
H	2.922655	-1.337958	3.834112
H	1.742250	-1.977350	2.674546
H	1.191817	-1.089566	4.100749
C	5.614720	-0.848098	1.287656
C	3.190490	-1.637532	-0.652801
C	4.463761	1.325194	-0.783397
C	5.708789	1.005588	-1.651321
H	6.617651	0.899738	-1.044675
H	5.883522	1.849469	-2.345658
H	5.598715	0.099445	-2.259145
C	3.314343	1.687570	-1.745914
H	3.024374	0.865536	-2.412426
H	3.618348	2.538015	-2.383998
H	2.427577	2.021850	-1.180531
C	4.774885	2.623032	-0.014025
H	5.633104	2.525868	0.660672
H	3.910343	2.954509	0.570802
H	5.016540	3.427838	-0.733993

C	3.976984	-1.942155	-1.954327
H	5.035318	-2.173307	-1.783020
H	3.917953	-1.115676	-2.676685
H	3.521028	-2.825529	-2.440275
C	1.745342	-1.330434	-1.090535
H	1.711926	-0.527006	-1.842775
H	1.105169	-1.039073	-0.249501
H	1.305287	-2.223667	-1.569314
C	3.142677	-2.948837	0.167938
H	4.143016	-3.362765	0.349147
H	2.571461	-3.709258	-0.394864
H	2.647470	-2.819986	1.137222
C	6.475014	-1.730413	0.342940
H	7.421017	-1.971094	0.863758
H	6.738646	-1.226687	-0.595467
H	5.993377	-2.686128	0.097782
C	6.535762	0.315677	1.735223
H	7.291371	-0.061570	2.448773
H	5.993867	1.124951	2.231774
H	7.082033	0.750528	0.886736
C	5.309823	-1.725919	2.524564
H	6.260734	-2.097457	2.949612
H	4.692169	-2.599091	2.280329
H	4.797409	-1.172024	3.316585
C	2.891484	3.999712	3.450380
C	0.899146	2.339845	5.376053
C	4.014850	1.732548	5.462626
C	4.374484	4.097777	3.021448
H	4.725900	3.200653	2.502440
H	5.037042	4.270168	3.881318
H	4.508272	4.951954	2.331878
C	2.009061	4.175829	2.192120
H	2.148032	3.359846	1.468913
H	2.268086	5.124417	1.684015
H	0.940355	4.204366	2.432440

C	2.626620	5.211470	4.379005
H	3.199066	5.158451	5.316010
H	1.565300	5.330269	4.634937
H	2.939224	6.135613	3.856613
C	5.267797	1.312690	4.678647
H	6.039280	0.934202	5.375732
H	5.715765	2.143907	4.121958
H	5.045476	0.509834	3.972090
C	3.634199	0.527294	6.356006
H	4.527661	0.201451	6.921186
H	3.282618	-0.334680	5.770773
H	2.860378	0.777986	7.093272
C	4.451096	2.888542	6.400589
H	5.249506	2.515665	7.070683
H	3.642615	3.266582	7.036583
H	4.872757	3.737549	5.844881
C	1.100243	3.076144	6.721861
H	1.738589	2.516810	7.419585
H	0.113254	3.192258	7.209027
H	1.523020	4.082933	6.600188
C	-0.208791	3.072595	4.604439
H	-0.386530	2.595641	3.633744
H	0.017625	4.131878	4.431912
H	-1.149596	3.036266	5.184988
C	0.347960	0.931567	5.684310
H	1.057520	0.290376	6.220579
H	0.055933	0.425623	4.758456
H	-0.560411	1.022694	6.307986
C	-3.136080	4.086996	1.155116
C	-2.508163	4.239156	-0.247236
H	-1.768731	5.060341	-0.233283
H	-3.244048	4.469709	-1.029211
H	-1.974016	3.329606	-0.556563
C	-4.001314	5.352967	1.384378
H	-3.378574	6.241838	1.170533

H	-4.338484	5.445181	2.425370
H	-4.882756	5.404834	0.730643
C	-1.972534	4.133942	2.159283
H	-1.459512	5.111282	2.087779
H	-1.236904	3.349066	1.938451
H	-2.306117	4.011277	3.194819
C	-5.177158	2.117182	-0.308841
C	-5.367805	2.574899	2.909881
C	-4.247404	1.619787	-1.441242
H	-3.591913	2.416455	-1.817101
H	-4.849623	1.260213	-2.295697
H	-3.612310	0.786121	-1.112351
C	-5.885561	3.399483	-0.819667
H	-5.185547	4.193198	-1.110024
H	-6.586151	3.815858	-0.083705
H	-6.473975	3.141149	-1.720619
C	-6.289947	1.065732	-0.111187
H	-5.903977	0.116661	0.267157
H	-6.783454	0.863879	-1.080360
H	-7.067184	1.413273	0.583774
C	-6.558635	3.524819	2.603214
H	-6.254210	4.525670	2.276410
H	-7.148950	3.648383	3.530688
H	-7.239371	3.107686	1.847681
C	-6.018344	1.248282	3.361535
H	-5.285786	0.491026	3.661854
H	-6.663397	0.813411	2.588872
H	-6.656660	1.445926	4.243086
C	-4.634033	3.151245	4.146820
H	-4.266398	4.172861	3.985451
H	-3.786239	2.530255	4.461081
H	-5.343610	3.192321	4.994477
C	-3.077818	-2.493718	-0.649881
C	-4.653199	-2.821426	2.138647
C	-1.508761	-3.318416	2.035796

C	-5.794617	-1.832782	1.840871
H	-5.488002	-0.800038	2.019044
H	-6.659914	-2.040451	2.497863
H	-6.149082	-1.910688	0.806114
C	-5.141031	-4.208941	1.647595
H	-4.432180	-5.020734	1.850105
H	-5.365115	-4.211871	0.571542
H	-6.083613	-4.455225	2.173117
C	-4.511588	-2.897679	3.677381
H	-3.822891	-3.692078	3.994399
H	-5.498674	-3.125249	4.121315
H	-4.163717	-1.951674	4.114880
C	-4.446696	-2.057589	-1.217181
H	-4.718144	-1.038986	-0.922958
H	-5.257319	-2.731069	-0.908298
H	-4.407604	-2.079969	-2.322383
C	-2.870694	-3.964992	-1.091733
H	-1.867739	-4.347270	-0.859513
H	-2.996471	-4.029038	-2.189380
H	-3.606537	-4.645796	-0.640521
C	-2.007511	-1.634550	-1.355668
H	-0.987106	-1.884112	-1.047265
H	-2.169174	-0.567215	-1.140727
H	-2.075865	-1.766218	-2.451785
C	-1.853020	-4.825903	2.123417
H	-0.939882	-5.380561	2.412112
H	-2.205873	-5.248348	1.173456
H	-2.610087	-5.035658	2.892090
C	-0.245877	-3.172404	1.169169
H	0.583810	-3.727811	1.641060
H	0.054116	-2.120500	1.082902
H	-0.368029	-3.581182	0.158250
C	-1.102355	-2.861910	3.455286
H	-0.316795	-3.537499	3.842083
H	-1.931472	-2.871566	4.173411

H	-0.675630	-1.853421	3.434340

(SiiPrDis2)2Tl-P

Atomic Number	Coordinates (Angstroms) X	Y	Z
Tl	-0.137234	-0.546892	1.442387
Si	2.437724	0.173703	0.583859
Si	-2.723541	-0.066523	0.495005
C	3.274052	0.022412	2.338232
H	2.857931	-0.991452	2.589342
C	4.804587	-0.138497	2.425455
H	5.169143	-0.928634	1.749203
H	5.096943	-0.429958	3.457405
H	5.348458	0.792494	2.185995
C	2.756567	0.953724	3.452366
H	3.238057	1.942982	3.409756
H	2.983536	0.517247	4.440985
H	1.670054	1.100999	3.406597
C	-3.982497	-0.174689	1.997026
H	-4.218396	0.900530	2.128727
C	-5.316459	-0.877593	1.670174
H	-5.204403	-1.973215	1.682866
H	-5.722151	-0.593065	0.683624
H	-6.084501	-0.623132	2.427405
C	-3.438059	-0.650340	3.355837
H	-2.562558	-0.068978	3.685369
H	-3.116262	-1.708501	3.344155
H	-4.215115	-0.551912	4.140550
C	-3.042297	1.735867	-0.153678
H	-2.350158	1.872516	-1.005143
Si	-2.482914	3.113079	1.064924
Si	-4.763699	2.122469	-0.902094

C	-1.490460	4.404215	0.109382
H	-0.511717	4.007810	-0.202274
H	-1.301610	5.287690	0.744750
H	-2.011954	4.749776	-0.798904
C	-3.872186	4.036390	1.955774
H	-4.511735	3.377868	2.565329
H	-4.524273	4.609819	1.277809
H	-3.397936	4.759480	2.643013
C	-1.370777	2.509636	2.472361
H	-1.872852	1.790016	3.130558
H	-1.090815	3.380200	3.085940
H	-0.426538	2.070824	2.116736
C	-4.601644	3.738479	-1.880786
H	-3.782012	3.682832	-2.620396
H	-4.401837	4.606267	-1.228465
H	-5.535814	3.948301	-2.433804
C	-6.153331	2.332233	0.376340
H	-7.084967	1.890222	-0.019771
H	-6.355122	3.395095	0.586389
H	-5.944436	1.841388	1.338398
C	-5.448616	0.847644	-2.125929
H	-5.741367	-0.098672	-1.639166
H	-4.775778	0.607379	-2.962137
H	-6.369710	1.280740	-2.560680
C	-3.068389	-1.419392	-0.867913
H	-4.120826	-1.197564	-1.142783
Si	-3.248460	-3.260620	-0.330967
Si	-2.238710	-1.246150	-2.583488
C	-2.780842	-3.649238	1.454957
H	-2.675559	-4.739382	1.547542
H	-3.543829	-3.327926	2.178319
H	-1.828803	-3.204203	1.762567
C	-5.052557	-3.737387	-0.643673
H	-5.247436	-4.771386	-0.310909
H	-5.274450	-3.689654	-1.724238

H	-5.767978	-3.076882	-0.129257
C	-2.262830	-4.534561	-1.327419
H	-1.177243	-4.453628	-1.203626
H	-2.490768	-4.524884	-2.403162
H	-2.561890	-5.522334	-0.939662
C	-3.431630	-1.973514	-3.864703
H	-3.574070	-3.058868	-3.734893
H	-3.041174	-1.812499	-4.885984
H	-4.427811	-1.501486	-3.812515
C	-0.584918	-2.143606	-2.801193
H	-0.713672	-3.220592	-2.966852
H	0.104087	-2.014315	-1.953597
H	-0.078736	-1.732460	-3.690967
C	-1.885892	0.532556	-3.126778
H	-1.142166	1.021695	-2.478072
H	-2.777765	1.176175	-3.165947
H	-1.450404	0.505889	-4.142571
P	0.214025	-1.997285	3.904595
C	2.220550	1.969783	-0.229741
H	1.129660	2.106388	-0.040247
C	3.223182	-1.225189	-0.501600
H	2.604667	-1.202684	-1.419773
Si	2.172367	2.130289	-2.144555
Si	2.896246	3.555842	0.623987
C	3.781362	2.728904	-2.950694
H	4.376151	3.410063	-2.320154
H	3.533061	3.274703	-3.880053
H	4.434935	1.886263	-3.230241
C	1.677347	0.529130	-3.015045
H	0.911706	-0.018052	-2.446888
H	2.517614	-0.156213	-3.195113
H	1.233177	0.770088	-3.997876
C	0.791451	3.333463	-2.632929
H	0.653105	3.283773	-3.729378
H	0.992628	4.383177	-2.371433

H	-0.173253	3.052774	-2.176191
C	4.720600	3.482211	1.095685
H	4.984906	4.421310	1.616070
H	5.380025	3.395064	0.215269
H	4.958317	2.648327	1.774290
C	1.868479	4.021543	2.148860
H	0.942030	4.528992	1.833956
H	2.442420	4.740556	2.760729
H	1.587352	3.187695	2.798931
C	2.712172	5.119906	-0.439334
H	3.222513	5.093052	-1.415091
H	3.149839	5.956535	0.137949
H	1.652702	5.371974	-0.612251
Si	5.007452	-0.990495	-1.193187
Si	2.923093	-3.036524	0.051324
C	3.893997	-3.588880	1.569080
H	4.983621	-3.456769	1.492513
H	3.696646	-4.660482	1.736761
H	3.545096	-3.054280	2.473317
C	1.132075	-3.448634	0.505624
H	1.002620	-4.530334	0.403806
H	0.369988	-2.982560	-0.127672
H	0.941336	-3.201128	1.547741
C	3.331743	-4.156464	-1.420094
H	4.379257	-4.099209	-1.751132
H	2.693855	-3.906375	-2.283929
H	3.125202	-5.203958	-1.149345
C	6.315932	-2.181062	-0.508106
H	6.516104	-2.026170	0.565804
H	7.261786	-1.970332	-1.043212
H	6.085378	-3.246859	-0.659048
C	5.725868	0.727084	-0.883522
H	6.179654	0.805040	0.118459
H	4.972833	1.521021	-0.971383
H	6.519042	0.940041	-1.624638

C	4.969634	-1.278940	-3.068253
H	4.575188	-0.406605	-3.617242
H	4.357159	-2.152708	-3.346911
H	5.995508	-1.457778	-3.439885

(SiiPrDis2)-Tl-P-(SiiPrDis2)

Atomic Number	Coordinates (Angstroms) X	Y	Z
Tl	1.032909	-0.066579	0.649659
P	-1.582191	-0.047993	0.689570
Si	-3.904265	-0.010874	0.699803
C	-4.266070	-1.737998	0.095013
H	-3.601205	-2.349777	0.718194
C	-4.449166	1.694870	0.189323
H	-4.490326	2.234882	1.140066
C	-3.971561	-0.029086	2.804322
H	-5.037532	0.000780	3.040995
C	-3.280359	1.205230	3.399744
H	-2.235630	1.278798	3.082220
H	-3.293005	1.155976	4.494376
H	-3.786908	2.132747	3.121739
C	-3.316480	-1.300309	3.360354
H	-2.258473	-1.362078	3.084386
H	-3.805281	-2.208965	3.002796
H	-3.377121	-1.311816	4.454154
Si	-3.631041	-2.156683	-1.631098
Si	-5.945368	-2.484224	0.447905
Si	-3.123823	2.708526	-0.715539
Si	-6.179831	1.995504	-0.418015
C	-4.351155	-1.132527	-3.013513
H	-4.272397	-0.068889	-2.801064
H	-5.404358	-1.375224	-3.176680

H	-3.820349	-1.325966	-3.950872
C	-3.964164	-3.977608	-2.128430
H	-3.426137	-4.201991	-3.054816
H	-5.026538	-4.150480	-2.311115
H	-3.602283	-4.654705	-1.350242
C	-1.737084	-2.104668	-1.720447
H	-1.358851	-1.085910	-1.759615
H	-1.392702	-2.616149	-2.624785
H	-1.303483	-2.615229	-0.855775
C	-5.739405	-4.388889	0.708085
H	-5.733498	-4.917871	-0.245767
H	-6.581034	-4.764518	1.297661
H	-4.810752	-4.590493	1.248686
C	-7.219246	-2.288678	-0.916585
H	-7.282726	-1.260444	-1.270239
H	-8.209189	-2.581205	-0.552789
H	-6.985372	-2.924342	-1.774603
C	-6.736343	-1.991311	2.093127
H	-6.859628	-0.916453	2.198478
H	-6.139214	-2.355249	2.933272
H	-7.728361	-2.447431	2.173418
C	-2.247703	1.878744	-2.142662
H	-1.477209	2.544523	-2.545091
H	-2.938982	1.657512	-2.958459
H	-1.750447	0.962806	-1.836203
C	-3.714264	4.365013	-1.453807
H	-2.856554	4.890507	-1.886263
H	-4.138988	5.006991	-0.678469
H	-4.440932	4.203873	-2.253569
C	-1.822903	3.294325	0.558518
H	-1.072703	3.915142	0.059810
H	-1.314516	2.458865	1.037489
H	-2.311494	3.893451	1.332520
C	-6.501170	1.605911	-2.213860
H	-5.730215	2.028491	-2.863792

H	-7.463067	2.024742	-2.526465
H	-6.545974	0.531377	-2.387559
C	-6.647473	3.861022	-0.188587
H	-6.404822	4.434592	-1.084526
H	-6.126113	4.273574	0.679104
H	-7.725484	3.948159	-0.023305
C	-7.515365	1.204161	0.654477
H	-8.475318	1.704677	0.491906
H	-7.263357	1.301064	1.713951
H	-7.663446	0.157491	0.406200
Si	3.717695	-0.073010	0.562205
C	4.229846	1.530001	-0.326134
H	5.269808	1.424128	-0.632975
C	4.602401	-1.714532	0.156184
H	4.924422	-2.083544	1.135466
C	4.057057	0.032611	2.647038
H	3.671983	1.014673	2.925885
C	5.545298	-0.088022	2.993529
H	5.709641	0.187391	4.041261
H	6.168406	0.560130	2.379334
H	5.913129	-1.111607	2.870419
C	3.276711	-1.037899	3.421933
H	3.451174	-0.924781	4.498050
H	3.589678	-2.048129	3.144731
H	2.199110	-0.959374	3.266449
Si	6.238200	-1.574165	-0.755908
Si	3.492881	-3.128358	-0.408019
Si	3.295662	1.866894	-1.975187
Si	4.192442	3.125070	0.726225
C	7.272961	-3.183676	-0.579518
H	7.122946	-3.623077	0.410125
H	8.335054	-2.945646	-0.694457
H	7.013889	-3.906050	-1.354875
C	7.446434	-0.323302	0.020092
H	7.712642	-0.642513	1.031414

H	7.054471	0.690778	0.063641
H	8.366179	-0.296908	-0.573188
C	6.123035	-1.218501	-2.586032
H	5.816737	-0.188114	-2.771596
H	5.421168	-1.892927	-3.083281
H	7.101147	-1.354495	-3.058680
C	4.442038	-4.734411	-0.828439
H	5.141131	-4.992644	-0.029616
H	4.969568	-4.632247	-1.779467
H	3.727933	-5.557602	-0.932413
C	2.398540	-2.853296	-1.895212
H	2.984681	-2.585266	-2.776848
H	1.643356	-2.089304	-1.715866
H	1.861249	-3.776851	-2.136308
C	2.397387	-3.715729	1.043311
H	1.739325	-2.927888	1.407162
H	3.029569	-4.055700	1.868565
H	1.777049	-4.555944	0.716820
C	5.559902	3.155942	2.048283
H	5.777804	4.192309	2.325837
H	6.479450	2.712658	1.657917
H	5.252798	2.641428	2.959159
C	4.660480	4.681131	-0.288920
H	4.805085	5.525556	0.392364
H	3.879500	4.960227	-0.997133
H	5.598706	4.509547	-0.823776
C	2.560249	3.490745	1.577243
H	2.634475	4.432862	2.130047
H	2.291454	2.712699	2.294889
H	1.740685	3.595006	0.863622
C	4.527256	2.736841	-3.164321
H	4.644337	3.791537	-2.911755
H	4.151003	2.673854	-4.189673
H	5.503303	2.246828	-3.117830
C	2.755426	0.350055	-2.945250

H	3.527704	-0.414184	-2.953674
H	2.551684	0.625254	-3.985221
H	1.830898	-0.061311	-2.539314
C	1.755612	2.951318	-1.884367
H	1.979068	3.972787	-1.572188
H	1.011364	2.535491	-1.202737
H	1.294392	3.011559	-2.875741

Tl-P(Sii PrDis2)2

Atomic Number	Coordinates (Angstroms) X	Y	Z
P	0.999307	0.147504	0.722556
Tl	-1.320489	0.974477	1.835836
Si	0.813221	0.033951	-1.616310
Si	1.468597	-1.824640	1.903939
C	0.234520	-1.824235	-1.739956
H	0.647788	-2.174180	-0.786028
C	0.762025	-2.818482	-2.779163
H	1.858321	-2.869569	-2.767894
H	0.438866	-2.587528	-3.798575
H	0.380518	-3.828438	-2.544871
C	-1.293471	-1.972642	-1.607026
H	-1.801901	-1.823344	-2.568525
H	-1.728724	-1.262441	-0.888422
H	-1.549585	-2.988058	-1.258037
C	-0.026841	-3.049852	1.634122
H	0.051959	-3.297269	0.564702
C	-0.062374	-4.387151	2.395591
H	0.911033	-4.875081	2.496756
H	-0.735356	-5.093480	1.877327
H	-0.472641	-4.239506	3.400328
C	-1.410451	-2.410430	1.846658

H	-1.524467	-2.020246	2.874897
H	-2.211023	-3.156679	1.700292
H	-1.598514	-1.599975	1.131298
C	2.567858	0.408762	-2.371650
H	3.175335	-0.321309	-1.793899
C	-0.642807	1.183648	-2.226283
H	-1.441380	0.792137	-1.559086
C	3.061272	-2.583571	1.082226
H	2.812184	-2.326961	0.027660
C	1.468114	-1.360912	3.819056
H	0.365050	-1.311065	3.970814
Si	-1.499994	0.848473	-3.934943
Si	-0.830785	3.054731	-1.858374
Si	3.481789	2.069917	-1.906410
Si	3.073278	0.049684	-4.195880
C	-3.332009	0.489496	-3.609576
H	-3.875693	1.377187	-3.253856
H	-3.479405	-0.312525	-2.869261
H	-3.810363	0.164844	-4.550526
C	-1.483582	2.276649	-5.185146
H	-1.872776	3.232035	-4.808124
H	-2.139335	1.961290	-6.017156
H	-0.490581	2.463794	-5.619575
C	-0.870753	-0.606152	-4.959546
H	-1.198458	-1.578541	-4.569661
H	0.219112	-0.626762	-5.069876
H	-1.299245	-0.500287	-5.971602
C	2.192319	1.218398	-5.399985
H	2.883904	1.988666	-5.774798
H	1.350526	1.738191	-4.924704
H	1.797522	0.672238	-6.271859
C	2.886519	-1.700514	-4.891952
H	3.415411	-1.689126	-5.862198
H	1.858794	-2.025383	-5.086567
H	3.378246	-2.463176	-4.267395

C	4.944795	0.273041	-4.424648
H	5.308498	1.301390	-4.302413
H	5.190038	-0.042098	-5.454530
H	5.521478	-0.371012	-3.740614
C	3.528238	3.359318	-3.298277
H	3.802293	4.325250	-2.835494
H	2.572671	3.507624	-3.813523
H	4.289680	3.136934	-4.060761
C	5.306547	1.691953	-1.531905
H	5.595130	2.129733	-0.563368
H	5.959738	2.131732	-2.302635
H	5.529356	0.620142	-1.489425
C	2.832636	3.012835	-0.409073
H	3.690506	3.519698	0.067725
H	2.364690	2.360696	0.340195
H	2.110708	3.790764	-0.693018
C	-2.661636	3.535668	-2.019085
H	-3.062318	3.502185	-3.042141
H	-2.768888	4.573992	-1.656896
H	-3.303162	2.901321	-1.383753
C	-0.468261	3.573473	-0.070936
H	0.296973	2.967545	0.432893
H	-1.400247	3.558459	0.523566
H	-0.112117	4.619142	-0.057322
C	0.141068	4.208680	-2.998290
H	1.043700	4.602427	-2.509986
H	-0.497102	5.071637	-3.256441
H	0.446691	3.740197	-3.942359
Si	1.968024	-2.623044	5.217247
Si	1.988761	0.388157	4.491796
Si	3.312442	-4.475123	0.891589
Si	4.737352	-1.659608	1.227230
C	3.790391	-3.092823	5.214202
H	4.440520	-2.268945	5.540202
H	4.139964	-3.435097	4.235019

H	3.931011	-3.926453	5.924243
C	1.609529	-1.942921	6.955946
H	1.836510	-2.761440	7.661905
H	0.548276	-1.681173	7.094715
H	2.216816	-1.080292	7.257137
C	1.028486	-4.263778	5.372039
H	1.361458	-4.692597	6.334051
H	1.231635	-5.012037	4.599102
H	-0.062200	-4.126952	5.444491
C	0.718622	0.920790	5.808404
H	1.003983	0.662011	6.837074
H	-0.274815	0.476742	5.622595
H	0.599253	2.016508	5.764762
C	3.726921	0.385388	5.251897
H	4.469607	-0.107507	4.606941
H	3.774399	-0.100030	6.237709
H	4.054956	1.430643	5.384078
C	1.980410	1.902960	3.360494
H	2.442807	2.716668	3.951113
H	0.957566	2.231664	3.116505
H	2.522371	1.799402	2.417603
C	3.545196	-5.426882	2.505378
H	4.555792	-5.264624	2.912805
H	3.447147	-6.507114	2.299914
H	2.829414	-5.171400	3.295761
C	1.927036	-5.258916	-0.137189
H	2.339068	-6.127110	-0.680672
H	1.544798	-4.556104	-0.891503
H	1.072286	-5.614956	0.453210
C	4.858148	-4.912714	-0.112958
H	5.802278	-4.643468	0.383224
H	4.855341	-4.462399	-1.117916
H	4.857628	-6.009851	-0.243585
C	5.682719	-1.793282	-0.412890
H	6.417864	-0.972910	-0.457909

H	5.033197	-1.709816	-1.299304
H	6.246775	-2.731444	-0.501527
C	5.923868	-2.320617	2.540497
H	6.923211	-1.897772	2.334072
H	6.025908	-3.416574	2.537805
H	5.646382	-2.011582	3.557143
C	4.567900	0.179059	1.553726
H	5.555342	0.655842	1.422782
H	4.230543	0.393442	2.575249
H	3.858264	0.650971	0.862275

(Tbt)2Tl-P

Atomic Number	Coordinates (Angstroms) X	Y	Z
Tl	2.045353	1.594539	0.772233
P	-0.338954	1.742459	1.630793
C	2.814577	1.952166	-1.240774
C	4.182402	2.041925	-3.765019
C	3.462043	3.161332	-1.668658
C	2.783732	0.829091	-2.130618
C	3.442809	0.913973	-3.378470
C	4.172323	3.139384	-2.890987
H	3.386143	0.070783	-4.067583
H	4.710555	4.041563	-3.185347
C	2.738426	1.684884	2.836297
C	3.459845	2.798704	5.368531
C	4.022473	1.395989	3.407619
C	1.771149	2.379018	3.632310
C	2.137159	2.875227	4.903369
C	4.368753	2.042748	4.606688
H	1.368499	3.325034	5.533980
H	5.380454	1.906303	4.998719

C	2.036856	-0.476539	-1.835043
H	1.857106	-0.566433	-0.742299
C	3.393561	4.567038	-1.048609
H	3.849119	5.194151	-1.842889
C	4.898255	2.108676	-5.097806
H	4.780169	1.122219	-5.589804
C	5.014382	0.306898	2.979566
H	5.730511	0.274103	3.828396
C	0.263549	2.480753	3.297538
H	-0.149425	1.783794	4.035708
C	3.921526	3.382930	6.685733
H	5.020592	3.507460	6.605521
Si	2.961496	-2.108499	-2.287137
Si	0.271321	-0.475028	-2.617822
Si	3.979131	3.304897	-6.267574
Si	6.791805	2.319260	-4.960861
Si	1.660730	5.380178	-1.073368
Si	4.606708	5.153326	0.302270
Si	-0.922068	3.953207	3.652783
Si	-0.970348	-0.211745	2.619651
Si	3.747710	2.043444	8.031466
Si	3.304793	5.142301	7.046628
Si	4.283712	-1.445066	3.184380
Si	6.312460	0.614617	1.627867
C	2.787716	-2.620476	-4.103015
H	3.253797	-1.909051	-4.802826
H	1.745102	-2.771094	-4.419620
H	3.310317	-3.585486	-4.229395
C	2.246353	-3.526432	-1.253628
H	3.055909	-4.186952	-0.901676
H	1.550923	-4.141597	-1.844650
H	1.703299	-3.174328	-0.365094
C	4.820050	-2.011514	-1.991597
H	5.285284	-1.191867	-2.558718
H	5.275439	-2.960622	-2.325655

H	5.077506	-1.874101	-0.933731
C	-0.552692	-2.167729	-2.454741
H	-0.073958	-2.959587	-3.048059
H	-1.596659	-2.077917	-2.802742
H	-0.586575	-2.506694	-1.407266
C	0.361046	0.010057	-4.433746
H	0.650844	1.065097	-4.540231
H	-0.634275	-0.112325	-4.895096
H	1.075762	-0.591187	-5.013677
C	-0.911284	0.715885	-1.775522
H	-0.568744	1.757833	-1.797943
H	-1.103134	0.454520	-0.723862
H	-1.879234	0.676250	-2.306737
C	2.204672	2.698148	-6.432566
H	2.152560	1.635487	-6.720443
H	1.653108	3.282411	-7.188887
H	1.675768	2.808417	-5.472522
C	4.807486	3.300929	-7.962625
H	5.803572	3.772135	-7.937132
H	4.194359	3.860882	-8.689575
H	4.931724	2.275934	-8.350580
C	3.897176	5.077059	-5.624304
H	4.885975	5.504567	-5.400068
H	3.284985	5.138388	-4.710881
H	3.420160	5.719080	-6.385519
C	1.812887	7.084664	-1.879201
H	2.326550	7.817860	-1.239458
H	0.808209	7.484635	-2.101704
H	2.362278	7.028387	-2.834870
C	0.505553	4.361505	-2.143751
H	0.413993	3.341755	-1.750336
H	0.888970	4.284619	-3.174624
H	-0.500626	4.811722	-2.179732
C	0.935187	5.603686	0.644702
H	-0.065536	6.062041	0.589649

H	1.564648	6.265329	1.259842
H	0.828322	4.647267	1.178588
C	0.045937	5.505098	4.060674
H	0.955526	5.533278	3.446105
H	0.357903	5.516018	5.113444
H	-0.533458	6.418116	3.855781
C	-2.172834	4.308190	2.277044
H	-2.833535	5.130892	2.611577
H	-2.806090	3.428500	2.079239
H	-1.720241	4.597523	1.317827
C	-2.009080	3.405438	5.101934
H	-1.433967	3.139614	6.004161
H	-2.606726	2.521349	4.815791
H	-2.725383	4.200331	5.373365
C	-2.836425	-0.133711	2.379206
H	-3.191924	0.696449	3.015387
H	-3.371816	-1.045459	2.685037
H	-3.100439	0.128838	1.343815
C	-0.776012	-0.385213	4.507052
H	0.250726	-0.356034	4.900506
H	-1.176898	-1.395938	4.733794
H	-1.385453	0.325124	5.089928
C	0.016642	-1.609437	1.858437
H	1.062034	-1.394966	2.127246
H	-0.052226	-1.595272	0.760986
H	-0.234100	-2.611888	2.236364
C	2.007641	1.325037	8.049954
H	1.909566	0.578315	8.857064
H	1.779397	0.822824	7.096169
H	1.240996	2.099081	8.213565
C	4.974585	0.662573	7.636049
H	6.010304	1.044166	7.615535
H	4.767415	0.211727	6.653790
H	4.928903	-0.139058	8.392984
C	4.198313	2.737815	9.725540

H	5.197927	3.203440	9.703052
H	4.223759	1.930746	10.477615
H	3.481370	3.498351	10.073807
C	4.533272	5.962196	8.220677
H	5.563127	5.830946	7.844507
H	4.495346	5.554560	9.242021
H	4.342565	7.047356	8.281747
C	3.328131	6.141184	5.450670
H	4.362773	6.254826	5.085868
H	2.913595	7.151126	5.612288
H	2.748868	5.660213	4.653421
C	1.584666	5.159520	7.826530
H	1.234702	6.195404	7.976220
H	1.592257	4.667552	8.813409
H	0.831442	4.644206	7.210060
C	4.550727	7.045262	0.330012
H	4.680914	7.485801	-0.671992
H	5.375417	7.415866	0.963873
H	3.614314	7.440148	0.754901
C	6.354710	4.697886	-0.241120
H	6.573445	5.136446	-1.230096
H	6.517645	3.614439	-0.315092
H	7.095543	5.098849	0.471677
C	4.265244	4.585954	2.052008
H	3.223053	4.774916	2.347135
H	4.451914	3.520206	2.220216
H	4.907842	5.144032	2.753313
C	7.415465	1.463721	-3.405848
H	8.519108	1.458387	-3.384512
H	7.070915	0.418291	-3.348469
H	7.058567	1.982647	-2.504047
C	7.341063	4.123763	-4.886872
H	8.436766	4.176471	-4.761766
H	6.887920	4.654816	-4.034031
H	7.085866	4.676853	-5.805124

C	7.587313	1.494357	-6.460962
H	8.685972	1.478564	-6.356436
H	7.349337	2.013196	-7.402389
H	7.249861	0.448197	-6.561177
C	2.943058	-1.379856	4.503146
H	3.385007	-1.177665	5.492439
H	2.403683	-2.339957	4.566924
H	2.219233	-0.582121	4.293984
C	5.611545	-2.629644	3.828440
H	6.303901	-2.973924	3.045365
H	5.127854	-3.523715	4.259762
H	6.213398	-2.166601	4.629141
C	3.610684	-2.167560	1.583450
H	4.424014	-2.586802	0.971893
H	3.087736	-1.423913	0.964900
H	2.899348	-2.984182	1.784221
C	5.664366	0.838754	-0.110036
H	6.464010	0.600102	-0.828020
H	5.317055	1.854890	-0.326285
H	4.827240	0.167732	-0.334529
C	7.323203	2.113911	2.163542
H	8.123412	2.334058	1.436621
H	7.801777	1.923793	3.139886
H	6.704235	3.016684	2.265952
C	7.496636	-0.858564	1.581846
H	8.352362	-0.610462	0.929721
H	7.023076	-1.763047	1.167477
H	7.898304	-1.111512	2.576139

Tbt-Tl-P-Tbt

Atomic	Coordinates (Angstroms)		
Number	X	Y	Z

Tl	1.745338	-0.222270	0.498873
P	1.029557	1.485615	-1.003364
C	1.706473	1.405188	-2.701466
C	2.808102	1.410232	-5.330168
C	2.368604	2.583108	-3.195387
C	1.510105	0.275182	-3.569954
C	2.088688	0.301792	-4.851037
C	2.905161	2.543110	-4.497071
H	1.974433	-0.570751	-5.499077
H	3.428189	3.426772	-4.867445
C	1.877451	-0.167958	2.652658
C	2.325193	1.227152	5.088593
C	3.178562	-0.183387	3.248484
C	0.766742	0.439136	3.330231
C	1.038713	1.144897	4.524090
C	3.364994	0.526742	4.453135
H	0.203231	1.617967	5.043955
H	4.357010	0.528027	4.915906
C	0.685798	-0.933305	-3.165059
H	0.403225	-0.795196	-2.105172
C	2.491530	3.910803	-2.440241
H	3.007006	4.570624	-3.169104
C	3.431419	1.383967	-6.707180
H	3.272987	0.366228	-7.118611
C	4.392606	-0.975108	2.743959
H	5.050141	-1.028292	3.636350
C	-0.724560	0.314389	2.967543
H	-1.226434	0.603464	3.913905
C	2.577316	1.950788	6.390996
H	3.653228	1.824754	6.627761
Si	1.616368	-2.592975	-3.173001
Si	-1.031936	-0.917268	-3.998144
Si	2.423616	2.488180	-7.885593
Si	5.328290	1.543684	-6.646241
Si	0.868090	4.924980	-2.213746

Si	3.807562	4.084117	-1.059584
Si	-1.625463	1.605184	1.845855
Si	-1.384471	-1.469480	2.860915
Si	1.703558	1.059027	7.833136
Si	2.403732	3.837601	6.206652
Si	4.140968	-2.848452	2.429306
Si	5.653684	-0.087564	1.603304
C	1.370195	-3.616346	-4.739287
H	1.612683	-3.054505	-5.655207
H	0.331460	-3.972825	-4.830032
H	2.023583	-4.505677	-4.707533
C	0.999303	-3.649416	-1.732098
H	1.494871	-4.635901	-1.735784
H	-0.087622	-3.819964	-1.781310
H	1.206557	-3.183753	-0.755605
C	3.460128	-2.273202	-2.966121
H	3.895873	-1.857804	-3.887700
H	4.000662	-3.201527	-2.715367
H	3.645830	-1.538991	-2.168105
C	-2.153986	-2.122023	-3.070319
H	-1.839044	-3.171579	-3.189509
H	-3.190081	-2.044185	-3.443065
H	-2.173357	-1.895811	-1.990982
C	-1.012076	-1.362954	-5.831398
H	-0.207502	-0.832062	-6.364041
H	-1.966823	-1.051979	-6.291247
H	-0.890106	-2.441748	-6.012963
C	-1.751871	0.810214	-3.855904
H	-1.163846	1.520641	-4.457374
H	-1.745291	1.166355	-2.816377
H	-2.791508	0.832413	-4.225862
C	0.654631	1.835255	-7.865596
H	0.606669	0.782482	-8.191853
H	-0.000469	2.423471	-8.530832
H	0.236513	1.885442	-6.846998

C	3.127541	2.364397	-9.631409
H	4.114959	2.848940	-9.709049
H	2.458704	2.855039	-10.359246
H	3.245804	1.312366	-9.941247
C	2.384681	4.295146	-7.351085
H	3.391502	4.728037	-7.246488
H	1.866060	4.410777	-6.386843
H	1.836854	4.895080	-8.098446
C	0.876840	6.212398	-3.598085
H	1.750730	6.882129	-3.521006
H	-0.030845	6.839548	-3.564180
H	0.912112	5.728240	-4.588755
C	-0.713076	3.931430	-2.413157
H	-0.835584	3.155290	-1.642095
H	-0.758950	3.435035	-3.392137
H	-1.575087	4.618313	-2.335346
C	0.737205	5.870735	-0.580631
H	1.403342	6.746903	-0.554529
H	0.950103	5.250517	0.301407
H	-0.297420	6.242890	-0.476828
C	-0.562638	3.142296	1.655527
H	0.415046	2.939992	1.197357
H	-0.391512	3.607299	2.640135
H	-1.080998	3.880779	1.021137
C	-2.252098	0.931391	0.197952
H	-1.455199	0.510751	-0.429985
H	-2.708380	1.756852	-0.375844
H	-3.032441	0.168881	0.350280
C	-3.180044	2.155485	2.773044
H	-2.955626	2.520848	3.789773
H	-3.928036	1.351547	2.855700
H	-3.652105	2.986395	2.219032
C	-3.270931	-1.440760	2.966109
H	-3.621945	-0.866348	3.839187
H	-3.642145	-2.474422	3.078978

H	-3.749417	-1.017219	2.069826
C	-0.761205	-2.381315	4.385025
H	0.327490	-2.286380	4.494899
H	-1.009296	-3.455575	4.350779
H	-1.227074	-1.956739	5.291020
C	-0.934971	-2.404007	1.284182
H	0.116760	-2.726867	1.264542
H	-1.127137	-1.807240	0.378930
H	-1.548024	-3.318769	1.205430
C	-0.068192	1.651305	8.094533
H	-0.552528	1.063503	8.893488
H	-0.677571	1.532979	7.183895
H	-0.108397	2.711919	8.392111
C	1.678734	-0.787633	7.455858
H	2.695068	-1.166303	7.254141
H	1.065569	-1.006992	6.568335
H	1.266812	-1.357484	8.306427
C	2.699054	1.343228	9.409837
H	3.759629	1.077171	9.257814
H	2.314680	0.716371	10.232872
H	2.663170	2.392392	9.742560
C	2.846973	4.653974	7.849557
H	3.815098	4.291555	8.234659
H	2.084838	4.453420	8.620947
H	2.921816	5.748994	7.734989
C	3.618450	4.382310	4.872122
H	4.651643	4.083802	5.118406
H	3.607190	5.478542	4.743674
H	3.362926	3.926862	3.901464
C	0.677741	4.386949	5.689362
H	0.632516	5.489864	5.664955
H	-0.106653	4.035478	6.377167
H	0.433491	4.024448	4.679006
C	4.212119	5.932688	-0.913988
H	4.207108	6.449335	-1.888389

H	5.221846	6.049782	-0.482307
H	3.513739	6.460999	-0.247686
C	5.372777	3.255506	-1.708406
H	5.781865	3.837106	-2.552493
H	5.180410	2.236599	-2.070950
H	6.150400	3.204220	-0.927485
C	3.439492	3.532888	0.704346
H	2.450876	3.876209	1.038656
H	3.464501	2.445215	0.833229
H	4.194571	3.983677	1.372057
C	5.956433	0.679324	-5.093842
H	7.059855	0.687306	-5.059610
H	5.626116	-0.372525	-5.061574
H	5.582236	1.171029	-4.182716
C	5.924482	3.333473	-6.637272
H	7.025963	3.366221	-6.572046
H	5.527173	3.897580	-5.777920
H	5.630929	3.866070	-7.556834
C	6.040807	0.664572	-8.157336
H	7.141645	0.609808	-8.099123
H	5.779885	1.176000	-9.097308
H	5.660501	-0.369807	-8.223621
C	2.558100	-3.448469	3.239060
H	2.517923	-3.139777	4.296297
H	2.502384	-4.549907	3.202659
H	1.665015	-3.042467	2.746797
C	5.575648	-3.755126	3.259016
H	6.541620	-3.566378	2.766611
H	5.400096	-4.844831	3.244794
H	5.673000	-3.450256	4.315793
C	4.123138	-3.331929	0.605662
H	5.090304	-3.133284	0.118018
H	3.351233	-2.808806	0.019686
H	3.918786	-4.412075	0.506742
C	5.192048	0.063747	-0.213725

H	6.054171	0.497340	-0.748376
H	4.330138	0.710861	-0.433276
H	5.005195	-0.922925	-0.660918
C	6.032995	1.601814	2.342595
H	6.642266	2.202310	1.645682
H	6.606527	1.491513	3.278807
H	5.122069	2.170370	2.574427
C	7.262043	-1.082038	1.623914
H	8.052819	-0.498917	1.120229
H	7.175107	-2.040864	1.088373
H	7.608835	-1.294635	2.648533

Tl-P(Tbt)2

Atomic Number	Coordinates (Angstroms) X	Y	Z
Tl	6.175782	-0.260004	0.527463
P	3.645478	0.698352	0.847984
C	3.272232	1.626452	-0.811519
C	2.519178	2.489878	-3.513449
C	3.487077	2.994271	-1.254186
C	2.838122	0.688785	-1.848253
C	2.451355	1.144703	-3.122192
C	3.047883	3.364831	-2.557376
H	2.131264	0.402885	-3.856713
H	3.205833	4.401629	-2.855261
C	3.221528	1.355575	2.562443
C	3.098911	2.903109	5.009470
C	4.166018	1.112752	3.652768
C	1.997780	2.043835	2.944221
C	2.033342	2.875490	4.094558
C	4.088076	1.930341	4.797118
H	1.137814	3.454249	4.323303

H	4.821583	1.770418	5.593338
C	2.920355	-0.835883	-1.744381
H	3.180018	-1.102376	-0.704900
C	4.295769	4.173146	-0.644349
H	4.796181	4.559597	-1.557669
C	2.191195	2.908487	-4.935740
H	1.708933	2.037471	-5.423989
C	5.146533	-0.087306	3.892647
H	5.047551	-0.123845	4.998038
C	0.547271	1.814469	2.432493
H	-0.017784	2.166948	3.323376
C	3.091087	3.718145	6.291137
H	4.065241	3.532794	6.790172
Si	4.369877	-1.515593	-2.801566
Si	1.265406	-1.764332	-1.994432
Si	0.902264	4.295248	-5.151310
Si	3.807569	3.134725	-5.931818
Si	3.369931	5.808120	-0.219072
Si	5.890860	3.968809	0.345190
Si	-0.367555	3.042798	1.296928
Si	-0.147744	0.026356	2.560615
Si	1.843181	2.976661	7.529760
Si	3.117841	5.600503	6.021211
Si	4.407151	-1.856852	3.804193
Si	7.083149	0.031948	4.081064
C	3.941175	-1.950512	-4.587257
H	3.385355	-2.896031	-4.662765
H	4.881606	-2.072321	-5.151922
H	3.355032	-1.169788	-5.092464
C	5.003421	-3.097662	-1.983477
H	4.260971	-3.908701	-2.060444
H	5.218323	-2.958876	-0.910398
H	5.931312	-3.447233	-2.467671
C	5.784335	-0.253759	-2.912927
H	5.431747	0.779080	-2.772535

H	6.260808	-0.314136	-3.904871
H	6.591915	-0.447406	-2.186140
C	-0.127215	-0.685634	-1.350569
H	-1.024301	-1.286144	-1.128716
H	-0.396951	0.060938	-2.113529
H	0.161141	-0.140963	-0.445401
C	1.382864	-3.409919	-1.073812
H	1.985571	-4.136789	-1.643669
H	0.379304	-3.850393	-0.941233
H	1.836711	-3.303269	-0.077746
C	0.757115	-2.191561	-3.767722
H	0.828827	-1.339058	-4.461258
H	-0.303889	-2.499455	-3.743638
H	1.328291	-3.027350	-4.197385
C	-0.407985	4.162984	-3.814687
H	-0.807956	3.138221	-3.743897
H	-1.250100	4.844782	-4.024453
H	0.003284	4.424157	-2.831794
C	0.043941	4.054913	-6.816984
H	0.703503	4.243221	-7.677490
H	-0.816112	4.742499	-6.899689
H	-0.348360	3.027316	-6.912153
C	1.663792	6.022626	-5.101397
H	2.356071	6.192110	-5.942063
H	2.219817	6.208486	-4.168907
H	0.869809	6.786886	-5.167303
C	4.226931	7.235788	-1.125156
H	5.195300	7.522825	-0.691191
H	3.573021	8.125487	-1.093953
H	4.393131	6.991799	-2.189248
C	1.624123	5.824396	-0.916969
H	1.077712	4.894662	-0.738595
H	1.655020	5.990652	-2.003448
H	1.052369	6.655004	-0.471532
C	3.393135	6.235502	1.618490

H	3.134784	5.388861	2.268186
H	2.678634	7.048028	1.830864
H	4.390146	6.586211	1.927256
C	0.162741	4.765361	1.826781
H	1.242538	4.924427	1.775936
H	-0.152141	4.928507	2.870340
H	-0.329938	5.535044	1.212430
C	-0.271758	2.753372	-0.547657
H	0.746611	2.658076	-0.946596
H	-0.778895	3.580133	-1.067965
H	-0.820047	1.835967	-0.802562
C	-2.213378	3.046559	1.750410
H	-2.380515	2.849447	2.823490
H	-2.816091	2.328308	1.177476
H	-2.614690	4.054190	1.541942
C	-1.862776	-0.155652	1.777078
H	-2.639439	0.385232	2.337277
H	-2.120312	-1.229815	1.811537
H	-1.913794	0.156647	0.725015
C	-0.464071	-0.248238	4.408042
H	0.389579	0.036269	5.039219
H	-0.706244	-1.302510	4.625983
H	-1.325446	0.364361	4.728165
C	0.890906	-1.366443	1.838674
H	1.434521	-1.916933	2.613698
H	1.634232	-0.989405	1.123869
H	0.233539	-2.082044	1.322091
C	0.062598	3.444554	7.121352
H	-0.634633	2.941983	7.813779
H	-0.214165	3.145082	6.097983
H	-0.101576	4.530827	7.215111
C	2.029583	1.101708	7.483010
H	3.048528	0.799832	7.781733
H	1.851649	0.701250	6.475247
H	1.318792	0.617809	8.174704

C	2.244163	3.535749	9.289552
H	3.320576	3.424333	9.507375
H	1.695087	2.912437	10.017184
H	1.973061	4.585210	9.479721
C	3.116198	6.510684	7.675857
H	3.874405	6.115143	8.371710
H	2.136845	6.454467	8.177932
H	3.339399	7.579444	7.511739
C	4.730950	6.009987	5.135708
H	5.595966	5.786330	5.784050
H	4.782230	7.078119	4.863514
H	4.844136	5.419791	4.215362
C	1.653318	6.228238	5.022873
H	1.709785	7.326219	4.922923
H	0.694486	5.986568	5.509220
H	1.633641	5.804749	4.010960
C	6.851743	5.602767	0.316856
H	6.990924	5.996217	-0.703273
H	7.856181	5.401483	0.731520
H	6.400489	6.395300	0.932059
C	7.032262	2.813568	-0.632997
H	7.527317	3.393089	-1.431591
H	6.482277	2.005792	-1.139515
H	7.835073	2.373434	-0.016117
C	5.654021	3.537640	2.145543
H	4.675719	3.882266	2.503398
H	5.668186	2.460289	2.324294
H	6.429076	4.009744	2.767407
C	4.656440	1.460026	-6.052534
H	5.621523	1.543318	-6.582185
H	4.034169	0.732701	-6.601568
H	4.851166	1.045358	-5.054113
C	4.987962	4.348779	-5.099788
H	5.883818	4.487172	-5.729999
H	5.321738	3.974329	-4.118767

H	4.531365	5.339246	-4.947364
C	3.467791	3.718292	-7.696777
H	4.409191	3.704240	-8.273947
H	3.065516	4.742652	-7.747648
H	2.756608	3.050627	-8.212073
C	2.793149	-1.738476	4.760466
H	3.021459	-1.566195	5.826029
H	2.190642	-2.658848	4.692765
H	2.181017	-0.897361	4.418260
C	5.470568	-3.043477	4.830167
H	6.495981	-3.188496	4.460874
H	4.975507	-4.031050	4.812104
H	5.527296	-2.733201	5.885995
C	4.209821	-2.696615	2.129200
H	5.193384	-2.959995	1.700644
H	3.660819	-2.086782	1.401010
H	3.659944	-3.644276	2.264752
C	8.106876	-1.286260	3.159559
H	7.530507	-2.161627	2.824947
H	8.895608	-1.658144	3.837369
H	8.616231	-0.869327	2.274647
C	7.879401	1.692195	3.690026
H	8.927346	1.641140	4.035841
H	7.387181	2.520855	4.220104
H	7.894556	1.937180	2.618122
C	7.423030	-0.250286	5.922789
H	8.497901	-0.102084	6.125860
H	7.164281	-1.272139	6.242578
H	6.863134	0.456516	6.560491

(Ar*)2Tl-P

Atomic	Coordinates (Angstroms)		
Number	X	Y	Z

Tl	-0.381222	-0.883967	8.385714
P	-1.854938	1.046290	9.138935
H	-0.098268	-0.847472	3.111214
C	0.354928	-0.426763	4.011370
C	1.511685	0.615999	6.354375
C	-0.004081	-0.975630	5.264207
C	1.275594	0.621009	3.911576
C	1.865324	1.113934	5.080546
C	0.537109	-0.422200	6.460036
H	1.544734	1.034352	2.935605
H	2.624118	1.896738	5.022259
C	-0.916886	-2.172795	5.173403
C	-2.637531	-4.346706	4.512767
C	-2.328846	-2.014351	5.231375
C	-0.368953	-3.428232	4.778752
C	-1.242325	-4.481152	4.449356
C	-3.159371	-3.109328	4.924846
H	-0.824536	-5.439520	4.129649
H	-4.242090	-2.975285	4.964762
C	2.290976	1.217617	7.498291
C	3.925240	2.550773	9.426463
C	1.858852	2.442427	8.092693
C	3.576888	0.698308	7.834588
C	4.346914	1.364685	8.810581
C	2.683897	3.075594	9.039787
H	5.324252	0.944941	9.064326
H	2.346751	4.006749	9.499843
C	-2.957854	-0.643584	5.461550
H	-2.220505	-0.005608	5.973848
C	-4.215679	-0.694560	6.338333
H	-5.033780	-1.254719	5.856419
H	-4.582369	0.325748	6.521029
H	-4.005102	-1.160630	7.311784
C	-3.275261	0.017834	4.101375

H	-2.372427	0.103541	3.478372
H	-3.690808	1.028430	4.250571
H	-4.017199	-0.581672	3.547261
C	1.136544	-3.638471	4.627022
H	1.635424	-2.840586	5.191500
C	1.619978	-4.981064	5.203697
H	1.237345	-5.837688	4.622357
H	1.311124	-5.105921	6.248594
H	2.719584	-5.028609	5.165482
C	1.573923	-3.518639	3.150152
H	1.321477	-2.534553	2.730034
H	1.079005	-4.290834	2.535775
H	2.664886	-3.658528	3.063106
C	-3.544728	-5.489217	4.071552
H	-2.900723	-6.374106	3.920314
C	-4.605546	-5.855526	5.127269
H	-5.303025	-5.019883	5.301640
H	-4.137232	-6.109883	6.090527
H	-5.201504	-6.721063	4.792022
C	-4.206591	-5.156819	2.715854
H	-3.446446	-4.914427	1.955902
H	-4.876012	-4.285718	2.811456
H	-4.806062	-6.008316	2.351267
C	0.578187	3.153857	7.655372
H	-0.131124	2.390459	7.299277
C	0.869917	4.114513	6.479667
H	1.605241	4.876372	6.787694
H	-0.054357	4.633400	6.174311
H	1.270488	3.585723	5.604945
C	-0.102497	3.944725	8.788912
H	-0.212398	3.348035	9.703364
H	-1.113082	4.244732	8.472040
H	0.456376	4.864781	9.028430
C	4.803968	3.306596	10.416111
H	4.154258	4.038808	10.927862

C	5.894442	4.095981	9.658060
H	5.444505	4.763540	8.906047
H	6.574655	3.405434	9.131621
H	6.496215	4.705331	10.353667
C	5.432322	2.402089	11.492439
H	6.131556	1.674516	11.049949
H	4.662787	1.839922	12.042603
H	6.002095	3.007013	12.217185
C	4.315746	-0.441235	7.116680
H	5.072086	-0.788335	7.841299
C	5.113381	0.118812	5.914117
H	5.787766	-0.656275	5.511646
H	5.720116	0.988572	6.211998
H	4.437418	0.430304	5.104195
C	3.515516	-1.678542	6.688789
H	4.214127	-2.489221	6.427607
H	2.905124	-1.464999	5.805243
H	2.854063	-2.049600	7.484183
H	-0.221280	-4.738742	12.184782
C	-0.577488	-3.744241	11.904021
C	-1.410668	-1.155329	11.187815
C	-0.114124	-3.169425	10.699234
C	-1.435846	-3.034014	12.753776
C	-1.810235	-1.726770	12.414517
C	-0.581723	-1.893008	10.304575
H	-1.770611	-3.476818	13.696080
H	-2.379214	-1.120243	13.121909
C	0.945002	-3.909539	9.946981
C	2.985014	-5.434417	8.702192
C	2.306171	-3.497686	10.063623
C	0.610878	-5.081982	9.227659
C	1.640838	-5.826445	8.620022
C	3.294878	-4.269618	9.430114
H	1.389651	-6.735076	8.064744
H	4.337803	-3.963009	9.513881

C	-1.730777	0.319833	11.013059
C	-2.769893	2.865437	11.910583
C	-3.131462	0.732799	10.634161
C	-0.967799	1.179667	11.964379
C	-1.474434	2.391072	12.349844
C	-3.553675	2.057530	11.129514
H	-0.883522	3.028393	13.011477
H	-4.542510	2.412450	10.827562
C	2.694431	-2.286751	10.919957
H	1.951165	-1.497409	10.728038
C	4.077501	-1.703452	10.595324
H	4.887769	-2.368810	10.937867
H	4.203596	-0.737548	11.106349
H	4.204304	-1.530850	9.521303
C	2.634516	-2.624371	12.426596
H	1.629880	-2.934087	12.739954
H	2.923181	-1.742783	13.022860
H	3.337635	-3.441645	12.659668
C	-0.834605	-5.562171	9.127351
H	-1.461072	-4.843733	9.675901
C	-1.335615	-5.577806	7.673337
H	-0.766486	-6.288006	7.052415
H	-1.245145	-4.587299	7.205900
H	-2.395691	-5.875498	7.634718
C	-1.012252	-6.937397	9.801919
H	-0.669112	-6.911704	10.848831
H	-0.433961	-7.717315	9.279301
H	-2.072917	-7.239854	9.792101
C	4.077913	-6.288313	8.070398
H	3.579225	-6.990964	7.379305
C	5.092205	-5.466033	7.252930
H	5.659124	-4.772334	7.894690
H	4.589428	-4.869602	6.477399
H	5.820771	-6.132413	6.761877
C	4.795712	-7.121898	9.154721

H	5.540670	-7.799220	8.703752
H	4.074241	-7.725732	9.727997
H	5.320219	-6.460589	9.864742
C	-4.265959	-0.277339	10.389248
H	-3.813485	-1.169506	9.928315
C	-5.319723	0.304368	9.426263
H	-5.981174	1.015277	9.947422
H	-5.957343	-0.496760	9.020773
H	-4.843964	0.830717	8.588713
C	-4.971714	-0.693823	11.698327
H	-4.314832	-1.269113	12.362192
H	-5.851240	-1.318413	11.468388
H	-5.324794	0.195343	12.246302
C	-3.223610	4.264217	12.300923
H	-4.262592	4.381473	11.945265
C	-2.358078	5.324961	11.584970
H	-2.398186	5.192253	10.493330
H	-1.302199	5.244429	11.891197
H	-2.705082	6.342984	11.830473
C	-3.218147	4.484622	13.827350
H	-3.826315	3.723064	14.341433
H	-3.624977	5.479823	14.073332
H	-2.196305	4.435222	14.237889
C	0.397430	0.708262	12.461262
H	0.501009	-0.353571	12.207202
C	0.539495	0.816294	13.990987
H	-0.272056	0.274108	14.501744
H	0.513539	1.864366	14.330196
H	1.501112	0.383780	14.314720
C	1.532182	1.450769	11.735208
H	2.511166	1.052249	12.046106
H	1.513894	2.529218	11.959960
H	1.453279	1.340399	10.646096

Ar*-Tl-P-Ar*

Atomic Number	Coordinates (Angstroms) X	Y	Z
Tl	-0.923650	-0.373664	-0.338367
P	1.097810	0.034130	0.760491
H	-0.565171	-0.044005	5.549818
C	0.173555	0.317979	4.830548
C	2.068896	1.241049	2.982198
C	-0.013943	0.005816	3.468624
C	1.282037	1.055219	5.277513
C	2.225707	1.520191	4.344831
C	0.963423	0.453723	2.529868
H	1.412641	1.258858	6.342348
H	3.098604	2.091445	4.669870
C	-1.249068	-0.767751	3.102417
C	-3.652035	-2.251825	2.687634
C	-1.202469	-2.182157	2.941872
C	-2.501121	-0.097674	3.021530
C	-3.673443	-0.854374	2.812023
C	-2.400051	-2.894815	2.739160
H	-4.640776	-0.343904	2.781867
H	-2.355751	-3.983627	2.648931
C	3.097400	1.684520	1.977230
C	5.116884	2.455379	0.115334
C	4.161619	0.790919	1.651381
C	3.039340	2.965712	1.350665
C	4.052830	3.316722	0.438773
C	5.145517	1.195343	0.728958
H	4.006415	4.308141	-0.022470
H	5.963391	0.511594	0.482641
C	0.111114	-2.951179	3.067863
H	0.922247	-2.212916	3.142236
C	0.400970	-3.833357	1.840996

H	-0.354436	-4.626250	1.719507
H	1.381710	-4.323643	1.948254
H	0.426091	-3.238476	0.918680
C	0.118219	-3.790041	4.363428
H	-0.078189	-3.160141	5.245338
H	1.094060	-4.285638	4.498617
H	-0.656251	-4.574811	4.329739
C	-2.626620	1.410041	3.231605
H	-1.611832	1.829338	3.269741
C	-3.373549	2.108283	2.082158
H	-4.416836	1.763635	2.001727
H	-2.888728	1.930779	1.111448
H	-3.398294	3.197052	2.246561
C	-3.309005	1.708026	4.584196
H	-2.769939	1.226077	5.413958
H	-4.346143	1.332515	4.593439
H	-3.339236	2.793820	4.774092
C	-4.956776	-3.031532	2.556858
H	-5.773109	-2.288179	2.524349
C	-5.019918	-3.851979	1.255706
H	-4.258750	-4.647643	1.254089
H	-4.842594	-3.213843	0.376311
H	-6.007018	-4.331612	1.146154
C	-5.197645	-3.934094	3.785617
H	-5.178978	-3.348570	4.718444
H	-4.421382	-4.713278	3.860913
H	-6.175607	-4.439435	3.711300
C	4.296658	-0.580792	2.314370
H	3.438955	-0.715999	2.988190
C	5.576126	-0.638331	3.174747
H	6.479757	-0.564410	2.546803
H	5.627167	-1.592048	3.725841
H	5.602906	0.187907	3.902523
C	4.270277	-1.741950	1.302299
H	3.330305	-1.752886	0.732881

H	4.360575	-2.703458	1.832074
H	5.106022	-1.675922	0.586490
C	6.237884	2.886794	-0.822997
H	6.835399	1.985375	-1.047598
C	7.169129	3.902828	-0.124898
H	7.564650	3.492846	0.817902
H	6.623440	4.830861	0.113890
H	8.020215	4.166645	-0.775653
C	5.718999	3.451848	-2.159322
H	5.170521	4.395910	-2.009087
H	5.039700	2.741972	-2.655754
H	6.560526	3.662556	-2.840316
C	2.010120	4.062660	1.651999
H	2.062129	4.739943	0.781997
C	2.464694	4.887207	2.877185
H	1.802826	5.757959	3.022431
H	3.496148	5.251526	2.745428
H	2.429492	4.275244	3.791121
C	0.531924	3.653690	1.792315
H	-0.097325	4.555061	1.707064
H	0.328188	3.193189	2.767898
H	0.224340	2.946976	1.010515
H	-2.248056	1.456687	-5.112735
C	-1.740525	0.644686	-4.586202
C	-0.356888	-1.412950	-3.212227
C	-1.668407	0.677229	-3.179044
C	-1.158927	-0.416726	-5.299131
C	-0.441810	-1.415428	-4.620507
C	-1.040548	-0.394255	-2.499354
H	-1.244982	-0.454524	-6.387878
H	0.060335	-2.211204	-5.175964
C	-2.187867	1.878328	-2.435286
C	-3.183252	4.210116	-1.139258
C	-1.362646	3.035382	-2.311131
C	-3.502143	1.889319	-1.897344

C	-3.975431	3.058357	-1.269135
C	-1.876044	4.171396	-1.657507
H	-4.996286	3.079688	-0.876586
H	-1.242917	5.058100	-1.570633
C	0.513868	-2.452093	-2.548294
C	2.200818	-4.515012	-1.537858
C	-0.044596	-3.675852	-2.095737
C	1.924299	-2.251294	-2.471412
C	2.734611	-3.284461	-1.961611
C	0.809343	-4.682047	-1.603811
H	3.815947	-3.131838	-1.917707
H	0.383721	-5.636687	-1.280935
C	0.039477	3.104305	-2.920008
H	0.260123	2.126915	-3.372205
C	1.130415	3.378589	-1.867606
H	0.970444	4.346644	-1.366048
H	2.123338	3.412871	-2.344806
H	1.154542	2.595504	-1.096305
C	0.084505	4.157877	-4.047936
H	-0.693279	3.965988	-4.804167
H	1.067356	4.147355	-4.547688
H	-0.078094	5.173578	-3.650111
C	-4.435332	0.692147	-2.063648
H	-3.838397	-0.144895	-2.456099
C	-5.058963	0.233131	-0.732669
H	-5.698791	1.014613	-0.291920
H	-4.287542	-0.017485	0.011504
H	-5.684909	-0.660313	-0.890125
C	-5.521574	1.010622	-3.112144
H	-5.063222	1.309648	-4.067918
H	-6.165098	1.839509	-2.773348
H	-6.161574	0.131010	-3.292438
C	-3.755763	5.472402	-0.503298
H	-4.721337	5.193711	-0.044979
C	-2.856806	6.041340	0.611077

H	-1.901687	6.413900	0.205822
H	-2.625815	5.276773	1.367901
H	-3.355317	6.888026	1.111750
C	-4.042992	6.546366	-1.575692
H	-4.519971	7.432227	-1.122752
H	-4.710581	6.155372	-2.359936
H	-3.108787	6.874900	-2.060513
C	-1.537430	-3.962262	-2.226689
H	-2.040782	-3.017090	-2.482076
C	-2.163101	-4.478335	-0.920342
H	-1.720429	-5.436586	-0.603941
H	-3.243113	-4.641716	-1.056381
H	-2.025483	-3.762272	-0.096687
C	-1.783572	-4.945295	-3.389990
H	-1.352702	-4.557401	-4.326197
H	-2.863325	-5.108894	-3.542911
H	-1.314695	-5.921297	-3.181795
C	3.090647	-5.668446	-1.083858
H	2.420919	-6.461562	-0.706292
C	4.042974	-5.279358	0.061821
H	3.487666	-4.869410	0.917945
H	4.770086	-4.518086	-0.262168
H	4.611903	-6.160177	0.404190
C	3.880008	-6.255223	-2.274482
H	3.202781	-6.563313	-3.086925
H	4.465774	-7.134725	-1.956951
H	4.582457	-5.510568	-2.683981
C	2.594923	-0.987328	-3.013893
H	1.805313	-0.258106	-3.248251
C	3.342337	-1.311510	-4.326070
H	2.672752	-1.774065	-5.067503
H	4.170818	-2.013990	-4.134821
H	3.771020	-0.394796	-4.764264
C	3.551927	-0.319585	-2.011331
H	3.955948	0.612281	-2.436617

H	4.407709	-0.967345	-1.765254
H	3.043344	-0.066067	-1.072941

Tl-P(Ar*)2

Atomic Number	Coordinates (Angstroms) X	Y	Z
Tl	2.859563	-3.105636	7.146545
P	0.491560	-1.908714	8.246873
H	0.494043	-0.527994	3.674168
C	0.977239	-0.272283	4.619228
C	2.001084	0.591619	7.106747
C	0.392951	-0.742950	5.812004
C	2.138063	0.506314	4.632498
C	2.601421	0.952069	5.872749
C	0.974284	-0.429621	7.109789
H	2.617907	0.826875	3.704389
H	3.419905	1.675009	5.911425
C	-0.866868	-1.555018	5.619346
C	-3.274722	-3.003536	5.116851
C	-2.138703	-0.952064	5.866883
C	-0.822889	-2.846443	5.025055
C	-2.023057	-3.550538	4.808005
C	-3.307193	-1.688846	5.615365
H	-1.983603	-4.556131	4.377928
H	-4.273824	-1.224801	5.815726
C	2.383521	1.633983	8.149693
C	3.321930	4.042390	9.412043
C	1.683577	2.873297	7.995246
C	3.566192	1.630095	8.943363
C	3.991392	2.821104	9.567941
C	2.164341	4.034925	8.623812
H	4.902408	2.779803	10.171097

H	1.625959	4.976009	8.477928
C	-2.249055	0.515186	6.282257
H	-1.442963	0.721827	7.001408
C	-3.580502	0.889833	6.952929
H	-4.411651	0.891613	6.227602
H	-3.509260	1.903802	7.372529
H	-3.832428	0.207780	7.772520
C	-2.048149	1.414150	5.039569
H	-1.050341	1.291632	4.598467
H	-2.178800	2.475541	5.304209
H	-2.798185	1.160249	4.271833
C	0.476280	-3.479491	4.533939
H	1.302508	-2.826418	4.861464
C	0.723167	-4.894923	5.086510
H	-0.054625	-5.600145	4.754683
H	0.723466	-4.912538	6.186599
H	1.692745	-5.284602	4.733307
C	0.522110	-3.495331	2.990838
H	0.347270	-2.491310	2.576144
H	-0.256601	-4.162466	2.586425
H	1.501830	-3.854432	2.632829
C	-4.555194	-3.790383	4.869489
H	-4.255255	-4.806074	4.554299
C	-5.405801	-3.927022	6.147993
H	-5.740317	-2.941418	6.509953
H	-4.830568	-4.400672	6.957904
H	-6.304604	-4.536881	5.954794
C	-5.377241	-3.166757	3.721306
H	-4.773127	-3.088101	2.803041
H	-5.716577	-2.152155	3.988424
H	-6.270703	-3.775689	3.500862
C	0.478857	3.036035	7.065458
H	0.211581	2.050373	6.671179
C	0.836281	3.928803	5.857983
H	1.063716	4.959511	6.176828

H	-0.008056	3.969965	5.150890
H	1.713428	3.536613	5.322399
C	-0.754016	3.581622	7.805046
H	-1.075611	2.897381	8.600365
H	-1.592152	3.709606	7.102326
H	-0.559416	4.565853	8.261723
C	3.845957	5.350046	9.994150
H	2.982847	6.036305	10.067361
C	4.867305	5.985538	9.022928
H	4.427926	6.123943	8.022261
H	5.750656	5.333639	8.915745
H	5.208199	6.966911	9.394920
C	4.455614	5.208288	11.400953
H	5.378822	4.606400	11.381555
H	3.754136	4.729945	12.102057
H	4.721124	6.200123	11.802768
C	4.459208	0.418744	9.188325
H	5.304789	0.793109	9.791534
C	5.084827	-0.173894	7.913025
H	5.788510	-0.983215	8.170805
H	5.635645	0.596066	7.348809
H	4.321756	-0.578234	7.233652
C	3.742057	-0.632150	10.043833
H	4.437759	-1.433699	10.324671
H	2.887560	-1.073370	9.511084
H	3.345595	-0.183562	10.967152
H	1.365341	-4.562055	12.349980
C	1.011575	-3.584943	12.016193
C	-0.060659	-1.172140	11.072347
C	1.097315	-3.284248	10.642232
C	0.439203	-2.700256	12.935626
C	-0.117632	-1.520690	12.441151
C	0.610253	-2.018843	10.133802
H	0.370704	-2.956050	13.996120
H	-0.669413	-0.858802	13.111337

C	1.579956	-4.459811	9.824018
C	2.410629	-6.886055	8.551924
C	2.938064	-4.901589	9.910790
C	0.629049	-5.284120	9.141859
C	1.071694	-6.459920	8.508600
C	3.323451	-6.096798	9.266568
H	0.345266	-7.084010	7.983397
H	4.363469	-6.422205	9.344873
C	-1.011917	-0.055621	10.734816
C	-3.107832	1.862034	10.567762
C	-2.344403	-0.463723	10.438090
C	-0.733558	1.318816	10.997855
C	-1.797694	2.238294	10.910056
C	-3.358582	0.505171	10.338496
H	-1.605616	3.288588	11.118860
H	-4.381613	0.187503	10.116266
C	3.985276	-4.169236	10.751697
H	3.552693	-3.204739	11.049022
C	5.298815	-3.904621	9.988522
H	5.812294	-4.843374	9.726502
H	5.990144	-3.313623	10.611167
H	5.134050	-3.348329	9.052188
C	4.300875	-4.959080	12.041872
H	3.393211	-5.155080	12.629752
H	5.004294	-4.393357	12.675393
H	4.763721	-5.930599	11.801561
C	-0.869119	-4.983769	9.202281
H	-0.984331	-3.894944	9.259997
C	-1.667037	-5.448853	7.973783
H	-1.666376	-6.547156	7.866538
H	-1.287868	-5.001239	7.046685
H	-2.715888	-5.134893	8.081350
C	-1.470362	-5.597043	10.487133
H	-0.977777	-5.208636	11.389607
H	-1.362903	-6.695222	10.477830

H	-2.544292	-5.357894	10.553460
C	2.842861	-8.177727	7.870082
H	1.942018	-8.617105	7.406362
C	3.865891	-7.905580	6.747749
H	4.795220	-7.476037	7.157271
H	3.462466	-7.193368	6.010226
H	4.128995	-8.839418	6.223064
C	3.391915	-9.198603	8.888942
H	3.634616	-10.153463	8.393157
H	2.656270	-9.396830	9.684763
H	4.312558	-8.825554	9.367291
C	-2.751603	-1.936318	10.385303
H	-1.840668	-2.540756	10.435388
C	-3.479067	-2.321131	9.089388
H	-4.404198	-1.739828	8.943541
H	-3.766912	-3.383828	9.119957
H	-2.834610	-2.167519	8.214919
C	-3.597269	-2.292523	11.626993
H	-3.062091	-2.038894	12.555490
H	-3.822641	-3.371997	11.642910
H	-4.554743	-1.745192	11.625961
C	-4.244064	2.877607	10.532704
H	-5.100198	2.386911	10.035708
C	-3.899058	4.146050	9.730267
H	-3.607196	3.900348	8.698567
H	-3.064021	4.697804	10.191191
H	-4.765880	4.826787	9.693433
C	-4.676477	3.236192	11.971458
H	-4.951738	2.329866	12.534346
H	-5.539800	3.923361	11.966932
H	-3.849606	3.728084	12.510673
C	0.666813	1.834234	11.376373
H	1.351629	1.516405	10.574007
C	1.221091	1.279422	12.711791
H	1.491509	0.220535	12.652354

H	0.482307	1.401608	13.522175
H	2.126501	1.843501	12.993156
C	0.734603	3.369042	11.475292
H	1.778736	3.672167	11.622344
H	0.153245	3.731785	12.340541
H	0.370949	3.872284	10.571670
